# The SigD regulon of *Mycobacterium abscessus* determines cell envelope composition and antibiotic susceptibility

**DOI:** 10.1371/journal.pgen.1012286

**Published:** 2026-09-02

**Authors:** Sean R. Jones, Kelly Maune, Kelley Hurst-Hess, Pallavi Ghosh

**Affiliations:** 1 School of Public Health, University at Albany, Albany, New York, United States of America; 2 Division of Genetics, Wadsworth Center, New York State Department of Health, Albany, New York, United States of America; Tufts University School of Medicine, UNITED STATES OF AMERICA

## Abstract

A major determinant of the exceptional intrinsic resistance of M. abscessus is the lipid-rich cell envelope, yet the regulatory systems that remodel envelope-associated pathways remain poorly defined. Here, we determine the σ^D^ regulon in *M. abscessus* and establish its role in cell envelope homeostasis and intrinsic resistance to hydrophobic antibiotics. RNA-Seq analysis of a MabΔ*sigD* mutant identified 447 differentially expressed genes, while ChIP-Seq mapped 72 σ^D^ binding sites and defined a conserved promoter motif (GTAACA/G-N_16_-CGAT). Using a combination of σ^D^ binding, motif orientation and expression data, we identified a core set of directly regulated genes, distinct from what was previously observed in *M. tuberculosis*, many of which encode proteins involved in envelope-associated functions. These include loci involved in trehalose polyphleate (TPP) biosynthesis, the antigen 85 complex and peptidoglycan remodeling enzymes. Deletion of *sigD* resulted in a significant reduction in TPPs in the cell envelope and an increase in ethidium bromide accumulation. Consistent with these changes, loss of σ^D^ selectively sensitized *M. abscessus* to hydrophobic antibiotics, including rifampicin and tigecycline. Deletion of *mmpL10*, which is required for transport of TPP precursors, recapitulated the drug sensitivity of MabΔ*sigD*, implicating envelope composition as a key effector of the phenotype. Expression of the σ^D^ regulon further increased during starvation and in response to SDS, isoniazid, and ethambutol, mediated by degradation of RsdA, consistent with a role in stress-responsive envelope adaptation. Together, these findings demonstrate σ^D^ is active during logarithmic growth in rich media where it regulates the expression of envelope-associated genes that influence envelope permeability and basal level susceptibility to hydrophobic antibiotics; its activity further increases in response to cell envelope stress, presumably promoting envelope remodeling to counteract damage.

## Author summary

*Mycobacterium abscessus* is an increasingly important bacterial pathogen that causes difficult-to-treat infections because it is naturally resistant to many antibiotics. A major contributor to this resistance is its complex, lipid-rich cell envelope, which acts as a barrier that limits the entry of antibiotics. How this protective barrier is regulated and remodeled in response to environmental stress is not well understood. Here, we investigated the role of the alternative sigma factor D (σ^D^), a protein that controls the expression of specific groups of genes. We found that σ^D^ regulates numerous genes associated with the bacterial cell envelope, including genes involved in the production and transport of specialized surface lipids. Loss of σ^D^ altered the abundance of trehalose polyphleates and made bacteria more susceptible to several hydrophobic antibiotics. We further found that σ^D^ activity increases during nutrient starvation and when the cell envelope is disrupted. Our findings identify σ^D^ as part of a regulatory network that helps maintain the *Mycobacterium abscessus* cell envelope and influences its intrinsic resistance to antibiotics.

## Introduction

*Mycobacterium abscessus* is a rapidly growing non-tuberculous mycobacterium and a major cause of chronic pulmonary, skin, and soft tissue infections, particularly in individuals with underlying lung disease such as cystic fibrosis or bronchiectasis [1–3]. *M. abscessus* infections are among the most difficult bacterial diseases to treat, requiring prolonged multidrug regimens that are frequently ineffective and poorly tolerated [4,5]. This exceptional intrinsic resistance to antibiotics is driven by an impermeable lipid-rich cell envelope and a plethora of drug-and target-modifying enzymes that are induced in the presence of antibiotics [6,7].

The mycobacterial cell envelope is comprised of an outer mycomembrane (MM), peptidoglycan (PG) and arabinogalactan (AG) [8]. Long-chain (C60-90) α-alkyl, β-hydroxy fatty acids known as mycolic acids form the inner leaflet of the MM and are covalently linked to AG which in turn is attached to the PG layer. The outer leaflet of the MM is composed of mycolic acids linked to trehalose and other lipids and proteins such as glycopeptidolipids (GPLs), trehalose polyphleates (TPPs), and phosphatidyl-myo-inositol dimannosides (PIMs). [9]. Mycolic acid biosynthesis requires a coordinated action of a number of enzymes. Short-chain fatty acids synthesized by the fatty acid synthase I (FAS-I) system are iteratively elongated by the FAS-II system, consisting of the enzymes FabD, KasA, KasB, HadABC, and InhA [10]. Mycolic acids are then carboxylated by acyl-CoA carboxylase (AccD3), activated by acyl-AMP ligase (FadD2) and transferred to trehalose by Pks13 to form trehalose monomycolate (TMM) [11]. Once synthesized, TMM is transported across the plasma membrane by MmpL3 and incorporated into the cell envelope [12]. Additionally, mycolic acids are transferred from TMM either to AG to form mycolylarabilogalactan (mAG), or to another TMM to form trehalose dimycolate (TDM) by a set of secreted mycolyltransferases (the antigen 85 complex)**,** that are essential for mycomembrane assembly and maintenance [13]. Disruption of mycolic acid synthesis or transport profoundly compromises envelope integrity and viability [14].

Although *M. abscessus* (Mab) shares a conserved mycobacterial cell envelope architecture with *M. tuberculosis* (Mtb), their mycomembranes show differences in composition and dynamics that may have functional consequences for antibiotic susceptibility. For example, *M. abscessus* appears to have a lower abundance of oxygenated mycolic acids, which are key determinants of envelope permeability [15,16]. Moreover, in contrast to the *M. tuberculosis* mycomembrane which is dominated by long-chain mycolic acids and complex lipids such as phthiocerol dimycocerosates and sulfolipids, the *M. abscessus* mycomembrane is enriched in surface-exposed glycopeptidolipids (GPLs), which influence colony morphology, biofilm formation and disease progression [17,18]. Additional cell wall associated lipids such as trehalose polyphleates (TPP) - high molecular weight glycolipids structurally related to sulpholipids found in *M. tuberculosis*, and a glycosyl diacylated nonadecyl diol (GDND) have been reported in *M. abscessus* [19,20].

Despite the low permeability of the mycobacterial outer membrane, antibiotics reach the cytosol where they elicit a global reprogramming of gene expression mediated by transcription factors and alternate sigma (σ) factors. WhiB7 is the best studied transcription factor that induces genes involved in intrinsic resistance to ribosome targeting antibiotics [7,21,22]. Sigma factors are interchangeable subunits of RNA polymerase that confer promoter specificity and enable global remodeling of gene expression in response to defined cues [23]. In addition to the principal sigma factor, σ^A^, 12 alternate sigma factors belonging to groups II-IV have been identified in *M. tuberculosis* and 18 are known in *M. abscessus* [24,25]. In *M. tuberculosis*, σ^F^ induction is observed upon ethambutol (EMB), rifampin (RIF), streptomycin (STR) and cycloserine treatment [26], and σ^B^ and σ^F^ are induced upon vancomycin (VAN) exposure [27]. Furthermore, a MtbΔ*sigE* mutant was found to display VAN, RIF, STR, EMB, gentamicin (GENT) and isoniazid (INH) sensitivity, whereas MtbΔ*sigB* was INH and EMB sensitive [28].

The σ^D^ regulon has also been previously studied in *M. tuberculosis* [29,30]. Mtb*sigD* is transcribed throughout the logarithmic and stationary phases, but its activity is controlled primarily at the post-translational level by a transmembrane anti-sigma factor RsdA that sequesters σ^D^, keeping it inactive. Extracellular signaling and subsequent intramembrane cleavage of RsdA by a site 2 protease, Rip1, effectively liberates σ^D^ into the cytoplasm allowing it to associate with the RNAP core enzyme [31]. Although no growth defects were demonstrated in the MtbΔ*sigD* mutant in rich media, its expression was observed to increase during starvation and under INH exposure [29,32]. Strikingly, Δ*sigD* mutant strains of *M. tuberculosis* are significantly attenuated in mouse infection models, exhibiting reduced survival and an altered inflammatory response in the lung compared to the wild-type strain [29,30].

In *M. abscessus*, with the exception of σ^H^ that confers resistance to tigecycline (TIG) and amikacin (AMK) [22], much less is known about the role of the other 17 alternate sigma factors in adaptation to antibiotic stress. Here, we define the σ^D^ regulon of *M. abscessus* and demonstrate that activity of this sigma factor during exponential growth controls genes involved in biosynthesis/transport of cell envelope components which together influence envelope permeability and basal level susceptibility to hydrophobic antibiotics including RIF, CLA and TIG. In particular, we show that genes for TPP biosynthesis are σ^D^ regulated and deletion of either *sigD* or *mmpL10* results in increased drug sensitivity. Additionally, we also demonstrate that nutrient starvation and cell wall stress is sensed by membrane bound RsdA (regulator of σ^D^), leading to increased expression of the regulon which presumably counteracts the damage by remodeling the cell envelope.

## Results

### Determination of the σ^D^ regulon in *M. abscessus* using RNA-Seq and ChIP-Seq

**Fig 1:**
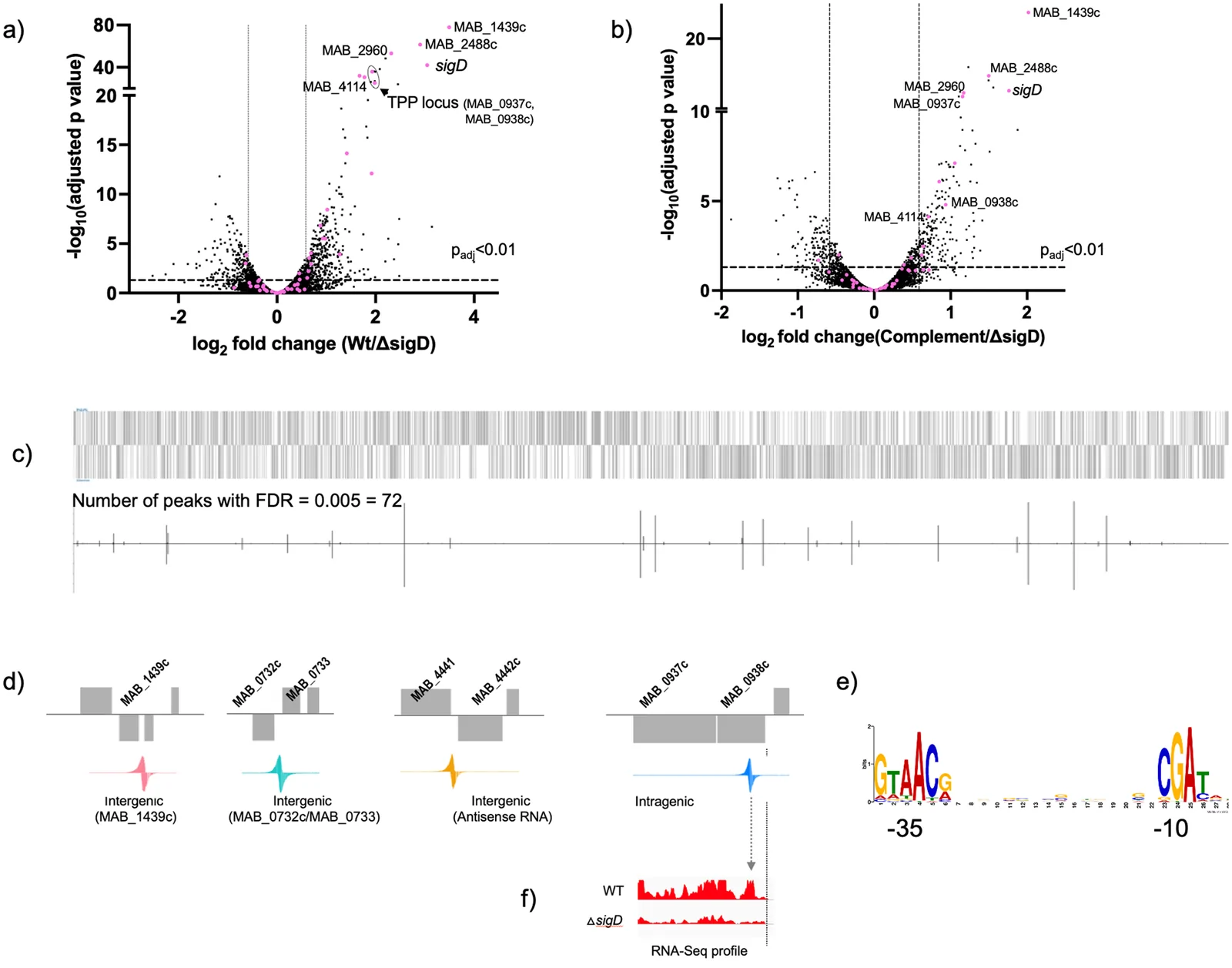
Determination of the *M. abscessus* σ^D^ regulon using RNA-Seq and ChIP-Seq. Volcano plots of differentially expressed genes between **a)** *M. abscessus* WT and MabΔ*sigD* and **b)** the *Mab*Δ*sigD::*p_*sigD*_*sigD* complemented strain and MabΔ*sigD,* determined by RNA-Seq. The horizontal dashed line represents an adjusted p-value of 0.01 and vertical lines represent a fold change of 1.5. Genes with a positive fold change represent genes underexpressed in the *ΔsigD* mutant, while a negative fold change indicates genes overexpressed in the mutant. Genes with direct binding sites for σ^D^ determined by ChIP-Seq are indicated in magenta. Genes strongly dependent on σ^D^ are labeled and MAB_0937c and MAB_0938c are circled as the TPP locus. **c)** ChIP-Seq was conducted using the *Mab*Δ*sigD::*p_*sigD*_*sigD*_3’**-**FLAG_ strain and peaks were called with a custom script using an FDR = 0.005. The distribution of peaks along the genome are shown. **d)** Representative examples of σ^D^ binding sites at different genomic locations relative to annotated open reading frames are shown. A representative intragenic σ^D^ peak within MAB_0938c coinciding with a known TSS suggesting a possible alternate start site is also shown. **e)** Sequence logo of the enriched motif in σ^D^ bound sites identified using MEME Suite 5.5.9 (E-value = 4.6e^-030^) showing consensus -35 and -10 elements. **f)** RNA-Seq reads for MAB_0938c in WT and MabΔ*sigD* visualized using IGV shows a large portion of reads starting within the MAB_0938c ORF corresponding to the binding site of σ^D^.

To determine the σ^D^ regulon of *M. abscessus* we generated an unmarked deletion of *sigD* (MAB_3724c) in a rough variant of the ATCC19977 type strain (CIP 104536^R^) using recombineering (S1 Fig). A complementing strain expressing *sigD* from its native promoter (Mab*ΔsigD::p*_*sigD*_*sigD*) was created by integration into the L5 attB site. Bacteria were grown to mid-exponential phase (A_600_ = 0.6) and changes in gene expression were determined using RNA sequencing (RNA-Seq) using the criteria of >1.5-fold change in the Mab*ΔsigD* strain as compared to wild-type (WT) bacteria (*p*_*adj*_ < 0.01). This identified 447 genes comprising the σ^D^ dependent regulon in *M. abscessus* (Fig 1a, S1 Data). Of these, 315 genes were downregulated and 132 genes were upregulated in Mab*ΔsigD*. Changes in gene expression between the complemented strain were also compared to Mab*ΔsigD* and identified 291 σ^D^ responsive genes (>1.5- fold change, *p*_*adj*_ < 0.01) (Fig 1b, S1 Data).

To determine the direct binding sites of σ^D^ we constructed a strain containing a 3X-FLAG tag at the 3’-end of *sigD* along with 200nt of its upstream regulatory sequence, integrated at the L5 attachment site of Mab*ΔsigD* and confirmed that it was fully functional (S2 Fig). The Mab*ΔsigD::p*_*sigD*_*sigD*_***3’-FLAG***_ strain was grown to mid-exponential phase (A_600_ = 0.6); DNA-nucleoprotein complexes were immunoprecipitated with anti-FLAG monoclonal antibodies followed by library preparation and Illumina sequencing. Using a previously published Python script, Peakcaller [33,34], we identified a total of 72 σ^D^ binding sites (FDR = 0.005) constituted by 44 intergenic binding sites (between annotated ORFs), and 28 intragenic sites (within annotated ORFs) (Fig 1c and 1d, Tables 1 and 2). Sequences corresponding to the Mabσ^D^ ChIP-Seq peaks were analyzed using MEME tools and a conserved motif containing clear consensus -35 and -10 elements, separated by 16 nucleotides, was identified in all 72 sites (Fig 1e). We then used a combination of peak location, motif orientation and gene expression changes between WT and Mab*ΔsigD* at all the 72 sites to determine the identity of genes regulated. Of the 44 intergenic peaks, 40 were found to be associated with the *M. abscessus* genes shown in Table 1, including *sigD* itself; 4 are likely involved in transcription of antisense RNAs. Of the 28 intragenic binding sites, 5 likely correspond to alternate start sites, and 18 are potentially involved in transcription of antisense RNAs (Table 2). Notably we observed that only MAB_0733, MAB_1097, MAB_1439c, MAB_1616, MAB_2488c, MAB_2960, MAB_3165c, MAB_4114 and MAB_4296c, were downregulated >2-fold in the Mab*ΔsigD* strain and were therefore strongly σ^D^ dependent. Most genes with σ^D^ binding sites in their promoter regions were either modestly downregulated or showed no significant difference in Mab*ΔsigD* (S1 Data), suggesting that these genes are not exclusively transcribed by σ^D^ and that their expression is likely compensated by additional sigma factors in the Mab*ΔsigD* strain. Interestingly, we observed a large intragenic σ^D^ binding site within MAB_0938c that coincided with the RNA-Seq profile as well as a previously described internal transcription start site (TSS); the expression of both MAB_0938c (*papA3*) and the operonic MAB_0937c (*mmpL10*) were significantly downregulated in Mab*ΔsigD* indicating a functional role of σ^D^ binding at this site (Fig 1d and 1f, Table 2) [35]. We also investigated if genes within the same transcription unit as the direct targets of σ^D^ were differentially expressed in wild-type *M. abscessus* when compared to Mab*ΔsigD* (RNA-Seq) and identified 9 additional genes within the σ^D^ regulon that are regulated directly by σ^D^ (Table 3). Therefore, binding of σ^D^ to 72 chromosomal locations suggests the regulation of 50 genes in *M. abscessus* (40 intergenic, 1 intragenic with a clear alternate start site, and 9 operonic genes).

**Table 1:** Intergenic σ^D^ binding sites determined by ChIP-Seq in *M. abscessus* ATCC/CIP^R^. Genetic location and coordinates of peaks, gene function and fold downregulation in Δ*sigD* mutant compared to WT and complemented (Δ*sigD* + p_sigD_*sigD*) strains (from RNA-Seq) are shown. Identity of genes regulated by σ^D^ binding was determined using a combination of peak location, the orientation of the conserved motif identified by MEME analysis as well as expression in Δ*sigD* and is also included. σ^D^ binding sites associated with a fold change >2 are highlighted in gray. Function of genes annotated as hypothetic proteins were verified using Uniprot(*).

	Coordinates	FAT Score	Genetic location	Gene Regulated	Function	Fold Change in WT/ΔsigD (RNA-seq)	p_adj_	Fold Change in Compl/ΔsigD (RNA-seq)	p_adj_
1	16074-16174	11.63	MAB_0016/MAB_0017	MAB_0017	YdeI/OmpD-associated family protein*	0.68	0.092	0.75	0.261
2	39670-39770	2.67	MAB_0039c/tRNA	MAB_0039c	FhaA domain containing protein*	1.15	0.335	1.12	0.502
3	58858-58958	2.2	end of MAB_0061	MAB_0062	Mycothione reductase	1.15	0.38	1.18	0.306
4	171520-171620	3.72	MAB_0174/MAB_0175	MAB_0175	Antigen 85 precursor	1.06	0.763	0.96	0.828
5	173926-174026	38.09	MAB_0176/MAB_0177	MAB_0177	Antigen 85-A/B/C precursor	0.64	1.01E-03	0.83	0.290
6	403368-403468	1.89	MAB_0401/MAB_0402	MAB_0402	Lipase/SGNH hydrolase family protein*	1.34	0.138	0.91	0.733
7	406156-406256	78.98	MAB_0405c-tRNA	MAB_0405c	secreted protein*	1.57	2.90E-04	1.40	0.015
8	411338-411438	42.09	MAB_0408/MAB_0409	MAB_0409	WhiB4	1.84	1.5E-07	1.80	8.13E-07
9	707814-707914	2.71	start of MAB_0706c	MAB_0706c	membrane protein*	0.75	0.21	0.60	0.020
10	737014-737114	23.05	MAB_0732/MAB_0733	MAB_0733	secreted protein*	3.79	7.72E-13	1.56	0.070
11	869215-869315	1.28	intragenic to MAB_0871c	MAB_0872	Probable cold shock-like protein B CspB	1.44	0.027	1.35	0.099
12	1110374-1110474	1.2	end of MAB_1096	MAB_1097	DNA sulfur modification protein DndE*	1.98	3.30E-05	1.91	1.55E-04
13	1129612-1129712	56.69	MAB_1118c/MAB_1119c	MAB_1118c	hypothetical	1.17	0.416	1.18	0.421
14	1164655-1164755	4.76	tRNA/MAB_1149	MAB_1149	transcriptional regulator TetR family	1.37	0.010	1.29	0.062
15	1446130-1446246	167.37	MAB_1439c/MAB_1440c	MAB_1439c	secreted protein*	11.27	1.23E-78	4.05	1.78E-24
16	1525246-1525346	1.5	MAB_1511/MAB_1512	MAB_1512	Probable fatty acid synthase Fas	0.98	0.916	1.06	0.781
17	1550245-1550345	3.72	tRNA/MAB_1530	MAB_1530	probable conserved lipoprotein (LppS)/ transpeptidase*	1.61	9.12E-05	1.07	0.741
18	1646235-1646335	21.42	MAB_1615/MAB_1616	MAB_1616	hypothetical/ secreted protein*	2.41	1.24E-04	1.64	0.071
19	1878224-1878324	1.59	end of MAB_1880c	MAB_1879c	malonyl coA -ACP transacylase (FabD)	0.95	0.813	1.10	0.622
20	2402669-2402769	3	MAB_2347c/MAB_2348	MAB_2348	possible long chain acyl-CoA-synthase/hydrolase*	0.83	0.173	1.06	0.782
21	2479345-2479474	128.92	MAB_2420c/MAB_2421c	MAB_2420c	sensor domain-containing protein*	0.84	0.408	0.90	0.703
22	2492182-2492282	29.37	end of MAB_2435	MAB_2436	APA family fibronectin-binding glycoprotein*	1.90	3.01E-06	1.56	3.42E-03
23	2543729-2543829	112.66	MAB_2488c/MAB_2489	MAB_2488c	hypothetical protein (9 kDa Antigen) (hemophore-related protein)	7.49	3.10E-62	2.82	2.09E-15
24	2924824-2924933	96.48	MAB_2871c/MAB_2872c	MAB_2871c	secreted protein*	1.97	3.16E-06	1.37	0.077
25	3014334-3014434	91.3	MAB_2959/MAB_2960	MAB_2960	hypothetical	4.98	5.66E-54	2.25	5.64E-13
26	3077960-3078060	1.57	MAB_3023c/MAB_3024	MAB_3024	DUF7455 domain-containing protein*	1.17	0.392	1.02	0.949
27	3212844-3212944	64.21	MAB_3165c/MAB_3166	MAB_3165c	L,D transpeptidase	2.03	3.58E-09	1.10	0.622
28	3249350-3249450	13.25	MAB_3206c/MAB_3207	MAB_3206c	Putative transcriptional regulator TetR	1.61	1.15E-03	1.36	0.071
29	3403232-3403332	87.58	MAB_3354/MAB_3355	MAB_3355	Putative secreted hydrolase	1.10	0.652	0.82	0.252
30	3434470-3434570	12.37	MAB_3384c/MAB_3385	MAB_3384c	putative ABC transporter ATP-binding protein	0.93	0.702	0.97	0.891
31	3781256-3781356	73.79	MAB_3724c/MAB_3725c	MAB_3724c	RNAP SigD	8.27	8.61E-43	3.39	2.73E-13
32	4125688-4125804	32.8	MAB_4080c/MAB_4081c	MAB_4080c	RpfD precursor (Resuscitation-promoting factor)/hydrolase*	1.06	0.81	0.98	0.925
33	4175106-4175206	163.79	MAB_4113/MAB_4114	MAB_4114	hemophore-related protein	3.42	2.67E-31	1.63	7.32E-05
34	4220193-4220293	1.06	end of MAB_4155c	MAB_4154c	MlaE family ABC transporter permease*	1.07	0.842	0.96	0.925
35	4301218-4301318	3.1	MAB_4230c/MAB_4231	MAB_4230c	F420-dependent glucose-6-phospahate dehydrogenase	0.77	0.063	0.85	0.361
36	4374700-4374800	172.95	MAB_4296c/MAB_4297c	MAB_4296c	DUF7159 family protein/ membrane protein*	3.20	9.96E-33	1.31	0.036
37	4423719-4423819	2.56	MAB_4343c/MAB_4344c	MAB_4343c	transcriptional factor WhiB	1.47	0.428	1.13	NA
38	4610094-4610194	1.37	MAB_4528c/MAB_4529	MAB_4528c	Conserved hypothetical protein	0.72	0.032	0.74	0.067
39	4620405-4620505	9.63	MAB_4537c/MAB_4538c	MAB_4537c	L,D transpeptidase*	0.78	0.047	0.96	0.816
40	4706463-4706563	1.16	MAB_4620c/MAB_4621c	MAB_4620c	DUF6578 domain-containing protein*	1.39	0.121	1.13	0.679
41	1118317-1118417	11.6	MAB_1102/MAB_1103	Antisense RNA	nc RNA reported in PMID: 27495169				
42	1612625-1612725	1.34	MAB_1582/MAB_1583	Antisense RNA					
43	4517525-4517625	109.83	MAB_4441/MAB_4442c	Antisense RNA	nc RNA reported in PMID: 27495169				
44	4950968-4951068	1.47	MAB_4838c/MAB_4839c	Antisense RNA					

**Table 2:** σ^D^ binding sites within annotated ORFs (Intragenic) determined by ChIP-Seq in *M. abscessus* ATCC/CIP^R^. Genetic location and coordinates of peaks, gene function and fold downregulation in Δ*sigD* mutant compared to WT and complemented (Δ*sigD* + p_sigD_*sigD*) strains (from RNA-Seq) are shown. Possible genes functions were assigned by using the orientation of the conserved motif identified by MEME analysis (**).

	ΔsigD Coordinates	ΔsigD FAT Score	Genetic location	Gene Regulated	Function	Fold Change in WT/ΔsigD (RNA-seq)	p_adj_	Fold Change in Compl/ΔsigD (RNA-seq)	p_adj_
1	934273-934373	36.35	intragenic to MAB_0938c	Alternate start in MAB_0938c	conserved polyketide synthase ass’d protein (PapA3)	3.95	6.24E-26	1.91	1.58E-05
2	112632-112732	11.9	intragenic to MAB_0112	Alternate start?	Hypothetical protein	1.34	0.125	1.20	0.421
3	4481738-4481838	10.39	intragenic to MAB_4400c	Alternate start?	Putative LysR transcriptional regulator	0.55	0.289	1.23	NA
4	4759904-4760004	7.81	intragenic to MAB_4680c	Antisense RNA**		n/a		n/a	
5	2780607-2780707	7.26	intragenic to MAB_2730	unknown		n/a		n/a	
6	2580728-2580828	6.16	intragenic to MAB_2533	unknown		n/a		n/a	
7	4136339-4136439	3.37	intragenic to MAB_4093	Alternate start?	Putative allophanate hydrolase	0.99	0.985	0.9	0.792
8	291148-291248	2.22	intragenic to MAB_0290	Antisense RNA**		n/a		n/a	
9	1211755-1211855	2.16	end of MAB_1196	Antisense RNA**		n/a		n/a	
10	2077554-2077654	2.14	end of MAB_2077	Antisense RNA**	nc RNA reported in PMID: 27495169	n/a		n/a	
11	44874-44974	2.09	intragenic to MAB_0047	Antisense RNA**		n/a		n/a	
12	4285562-4285662	1.97	intragenic to MAB_ 4217c	Antisense RNA**		n/a		n/a	
13	4181363-4181463	1.81	beginning of MAB_4116c	Antisense RNA**	nc RNA reported in PMID: 27495169	n/a		n/a	
14	4811604-4811704	1.78	Intragenic to MAB_4703c	unknown		n/a		n/a	
15	3298973-3299073	1.75	intragenic to MAB_3261c	Antisense RNA**		n/a		n/a	
16	4931102-4931202	1.72	intragenic to MAB_4817	Antisense RNA**		n/a		n/a	
17	1288554-1288654	1.67	start of MAB_1285c	Antisense RNA**	nc RNA reported in PMID: 27495169	n/a		n/a	
18	1336371-1336471	1.59	intragenic to MAB_1334c	Antisense RNA**	nc RNA reported in PMID: 27495169	n/a		n/a	
19	4925314-4925414	1.5	intragenic to MAB_4814	Antisense RNA**	nc RNA reported in PMID: 27495169	n/a		n/a	
20	13803-13903	1.48	start of MAB_0013c	Alternate start?	Probable arylamine n-acetyl transferase	0.98	NA	0.60	NA
21	4666971-4667071	1.48	end of MAB_4582c	Antisense RNA**		n/a		n/a	
22	3041086-3041186	1.45	intragenic to MAB_2982c	Antisense RNA**		n/a		n/a	
23	1843983-1844098	1.36	intragenic to MAB_1845c	unknown		n/a		n/a	
24	4018718-4018818	1.32	intragenic to MAB_3972c	Antisense RNA**		n/a		n/a	
25	3552048-3552148	1.28	intragenic to MAB_3510c	unknown		n/a		n/a	
26	777407-777507	1.23	intragenic to MAB_0782	Antisense RNA**		n/a		n/a	
27	4184954-4185054	1.17	intragenic to MAB_4120c	Antisense RNA**		n/a		n/a	
28	1384803-1384937	1.16	intragenic to MAB_1388c	Antisense RNA**		n/a		n/a	

**Table 3:** Expression of operonic genes regulated by direct σ^D^ binding.

Gene immediately downstream of SigD binding site	Operonic genes	Function	Fold Change in WT/ΔsigD (RNA-seq)	p_adj_	Fold Change in Compl/ΔsigD (RNA-seq)	p_adj_
Intragenic to MAB_0938c	MAB_0937c	MmpL10	3.81	1.34E-36	2.23	1.86E-12
MAB_1149	MAB_1150	Hypothetical protein	2.08	1.06E-03	1.45	0.181
	MAB_1151	Hypothetical protein	2.12	4.88E-07	1.28	0.216
	MAB_1152	Hypothetical protein	2.62	7.25E-14	1.67	4.36E-04
	MAB_1153	Hypothetical protein	1.79	1.04E-04	1.37	0.085
	MAB_1154	Hypothetical protein	1.8	2.23E-03	1.39	0.150
MAB_2960	MAB_2961	Putative menaquinone biosynthesis methyltransferase	5.49	1.27E-24	2.85	1.69E-08
MAB_4296c	MAB_4295c	Probable UDP-glucose 6-dehydrogenase (UdgA)	3.57	1.84E-16	1.27	0.306
MAB_4343c	MAB_4342c	Putative MutT/nudix family protein	1.97	1.76E-04	2.39	7.90E-07

**Fig 2:**
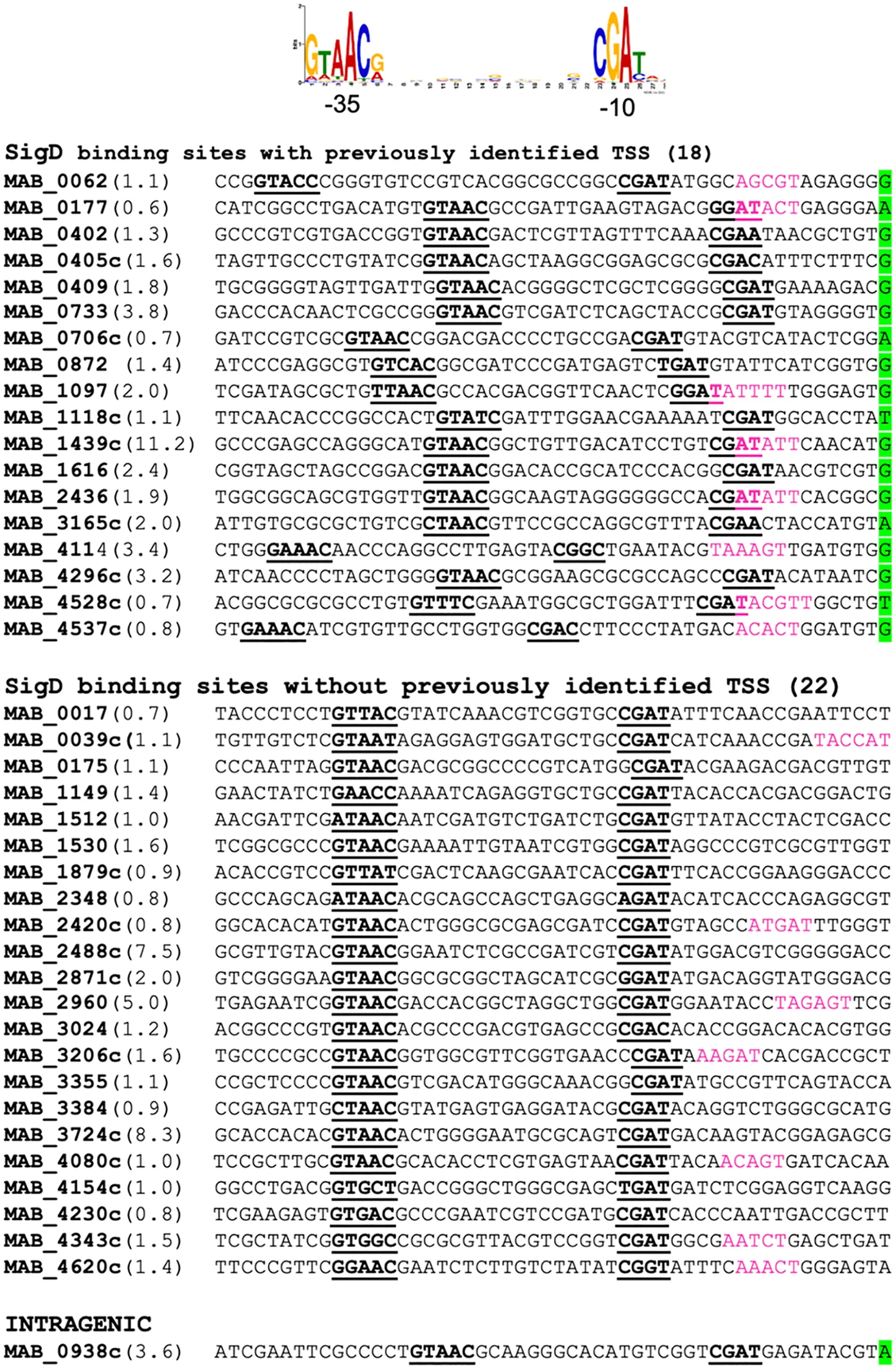
Sequence of σ^D^ promoters upstream of regulated genes. Sequence logo of the enriched motif in σ^D^ bound sites is shown. Location of -35 and -10 elements in σ^D^ promoters upstream of the regulated genes identified in Tables 1-2 are underlined and in bold. Approximately 50% of σ^D^ bound sites are associated with previously identified transcription start sites shown in green. Location of the σ^A^/σ^B^ −10 motif, ANNNT, with the conserved T appropriately positioned relative to the annotated transcription start site are in magenta. The fold-change in expression of each gene in WT compared to *Mab*Δ*sigD* is shown in parenthesis.

Lastly, we mapped previously identified transcription start sites (TSSs) located near σ^D^ binding sites (Fig 2) [35]. Approximately 50% of σ^D^ regulated promoters were associated with TSSs that were identified in cells grown in rich media (Fig 2). Several of these TSSs were linked to strongly σ^D^ dependent genes, including MAB_1439c, MAB_4114, and MAB_0733 suggesting that σ^D^ exhibits basal activity under these growth conditions. However, a number of σ^D^ bound promoter regions also contained a consensus −10 motif recognized by σ^A^/σ^B^ (TANNNT) at an appropriate distance from the annotated TSSs [34] (Fig 2). Many of the corresponding genes showed little or no dependence on σ^D^ for expression, suggesting that transcription from these promoters may be mediated by σ^A^ or σ^B^ associated RNA polymerase during logarithmic growth. These findings provide a potential explanation for the limited effect of *sigD* deletion on the expression of genes with σ^D^ promoters.

### The Mabσ^D^ regulon contains numerous cell envelope genes and Mab*ΔsigD* is deficient in TPPs

**Fig 3:**
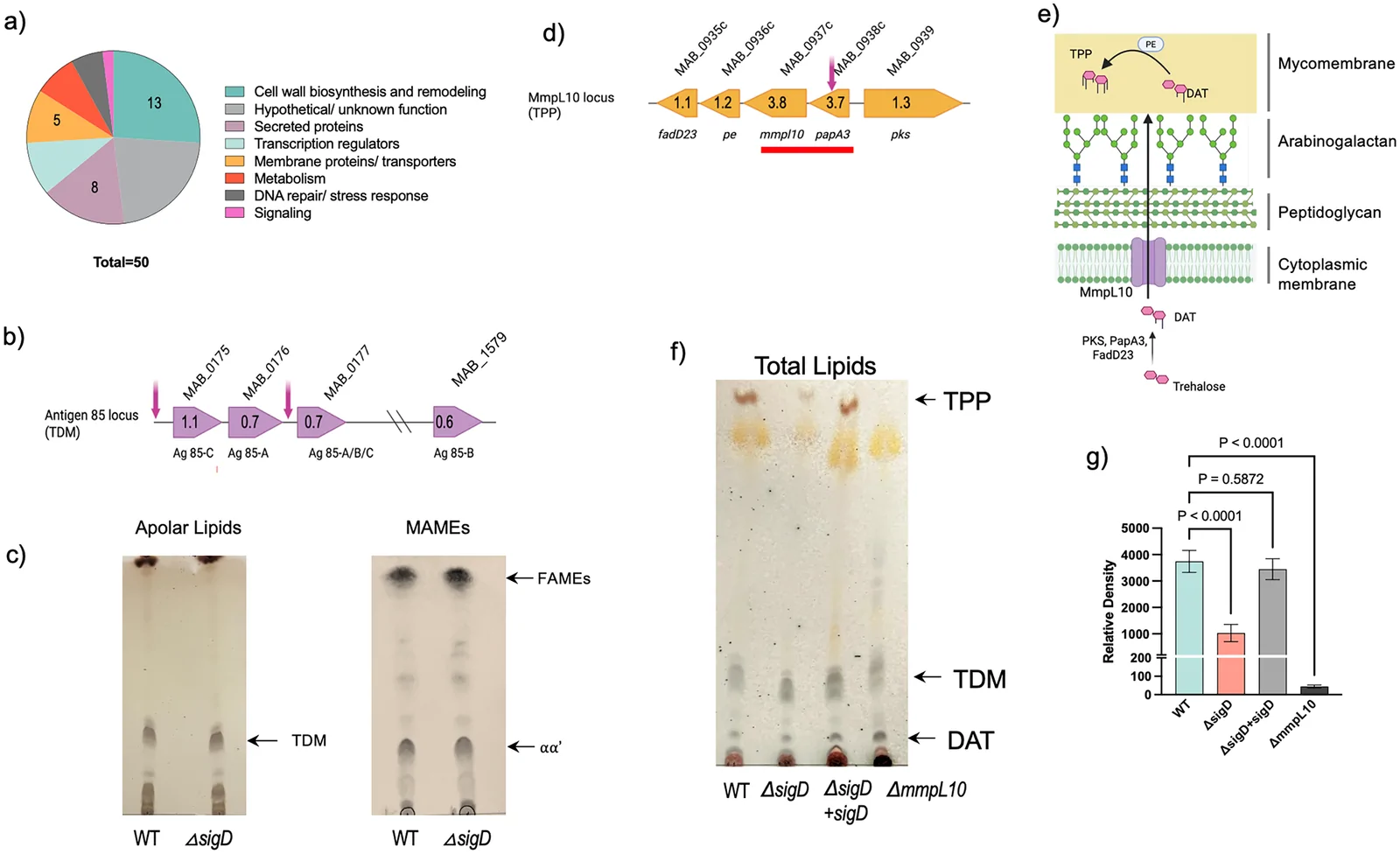
Mabσ^D^ regulon is enriched in cell envelope genes and a Mab*ΔsigD* mutant is deficient in TPPs. **a)** Pie chart of the functional categories of genes directly regulated by σ^D^. **b)** Schematic of the antigen 85 locus encoding proteins responsible for the production of the glycolipid TDM and mAG. Arrows indicate binding sites of σ^D^. Numerical values within each gene indicate the fold-change in expression of the gene in WT/ *Mab*Δ*sigD*. **c)** Thin Layer Chromatography of apolar lipids and mycolic acid methyl esters (MAMES) purified from WT and Δ*sigD* strains. Apolar lipids were resolved in CHCl_3_:MeOH:H_2_O (90:10:1 v/v) x2 and visualized by spraying with 10% H_2_SO_4_ in EtOH and charring at 120°C. MAMEs were resolved in petroleum ether:diethyl ether (95:5 v/v) x6 and visualized by spraying 5% phosphomolybdic acid and charring at 110°C. Data is representative of >3 biological replicates. FAME = Fatty acid methyl ester, αα’ = alpha and alpha prime mycolic acids. **d)** Schematic of the genes within the TPP locus responsible for TPP synthesis. Arrow indicates location of the σ^D^ binding site. The numerical values within each gene indicates the fold-change in expression of the genes in WT/ *Mab*Δ*sigD*; genes with a change in expression in the *ΔsigD* strain are underlined with a red bar. **e)** Schematic of the TPP biosynthesis pathway (Created in BioRender. Ghosh, P. 2026, https://BioRender.com/w7kkigq) showing the synthesis of diacyl trehalose (DAT) from trehalose in the cytosol, its transport to the cell envelope by MmpL10, and its subsequent conversion to TPP by the enzyme PE. **f)** Thin-layer chromatography analysis of total free lipids extracted from WT, Δ*sigD*, *Mab*Δ*sigD*::*p*_*sigD*_*sigD*, and Mab*ΔmmpL10* strains showing the migration of trehalose polypheates (TPP) and trehalose dimycolate (TDM) and is representative of 5 biological replicates. Total lipids were resolved using CHCl_3_/CH_3_OH (90:10 v/v) and visualized by spraying the plates with a 0.2% anthrone solution (w/v) in concentrated H_2_SO_4_ and charring. Images are representative of 5 independent experiments. TDM = Trehalose monomycolate, DAT = Diacyltrehalose and TPP = Trehalose polyphleate are annotated as per literature [19]. **g)** Quantification of TPP abundance from TLC plates by densitometric analysis using ImageJ. Data represent the mean relative spot density from five independent experiments (n = 5), with error bars indicating standard deviation (SD). Statistical significance was determined by one-way ANOVA followed by Dunnet’s multiple comparison test; P values are indicated.

Functional classification of genes directly regulated by σ^D^ revealed that many were associated with cell envelope-related processes. Approximately 27% of the σ^D^ direct targets were involved in cell envelope biosynthesis/transport and peptidoglycan remodeling; another 27% were either secreted or membrane proteins (Fig 3a). Thus, more than half of the direct target set comprises genes with known or predicted connections to the cell surface or envelope. This suggested a role of the σ^D^ regulon in cell envelope homeostasis and prompted us to examine envelope-associated phenotypes. Strikingly, σ^D^ binding sites were identified upstream of the antigen 85 complex genes involved in TDM and mAGP synthesis, as well as within MAB_0938c, a gene involved in TPP glycolipid biosynthesis (Fig 3b,3d-3e). While no change in expression of the ag85 genes was detected in Mab*ΔsigD* compared to WT bacteria*,* expression levels of both *MAB_0937c* and *MAB_0938c* were significantly downregulated in Mab*ΔsigD* (Tables 1 and 2). We therefore investigated the differences in cell envelope lipids in wild-type and Mab*ΔsigD* strains. Consistent with mRNA levels of ag85 genes, no differences were observed in the total amounts of TDM or methyl esters of mycolic acids (Fig 3c). In contrast, TPP levels were significantly lower in Mab*ΔsigD,* and restored in in the complemented strain (Fig 3f-3g). The identity of TPP was based on previous literature as well as by its absence in MabΔ*mmpL10* (Fig 3f-3g) [19].

### The Mabσ^D^ regulon is induced by nutrient starvation and agents that perturb the cell wall

**Fig 4:**
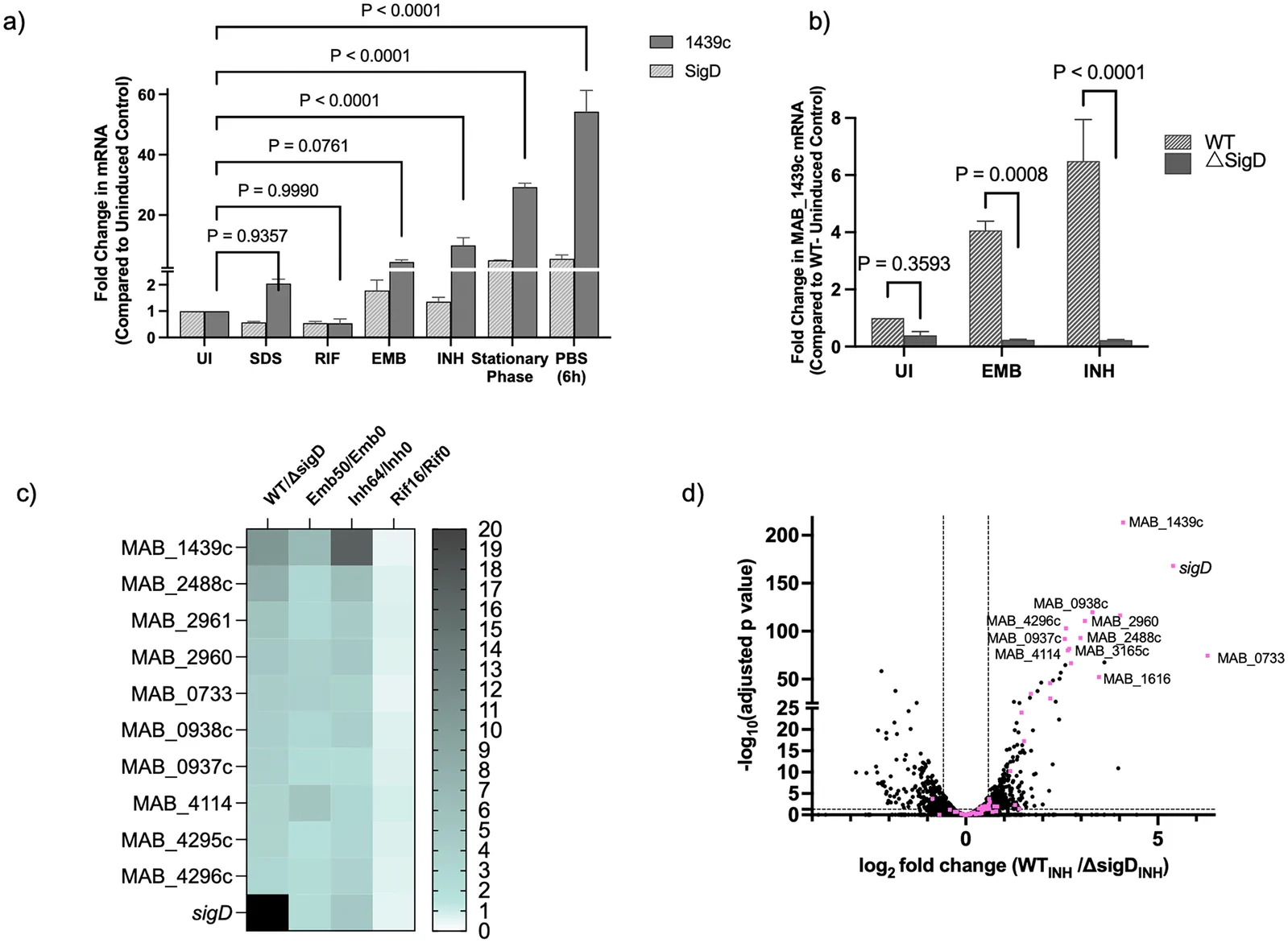
Expression of the σ^D^ regulon is induced by nutrient starvation and cell envelope stress. **a)** Change in relative mRNA expression of *sigD* and MAB_1439c in WT *M. abscessus* following exposure to 0.05% SDS, RIF 32μg/mL, EMB 70μg/mL, or INH 64μg/mL for 2h, nutrient starvation in PBS for 6h, or in stationary phase (A_600_ = 6.0). Transcript levels were measured using qRT-PCR, normalized to the endogenous control *sigA*, and relative expression was calculated using the 2^−ΔΔCt^ method and expressed as fold change relative to the uninduced control. Data are from 3 independent experiments and are presented as mean ± SD. Statistical analysis shown was performed for MAB_1439c with each treatment condition compared with the uninduced control using two-way ANOVA followed by Dunnet’s multiple comparison test. P values for these comparisons are indicated above the brackets. **b)** Change in relative mRNA expression of the σ^D^ dependent gene MAB_1439c in WT and *ΔsigD* when induced with EMB 70 μg/mL and INH 64 μg/mL for 2h. Transcript levels were measured using RT-qPCR, normalized to the endogenous control *sigA*, and relative expression was calculated using the 2^−ΔΔCt^ method and expressed as fold change relative to the wild-type uninduced control. Data represents mean ± SD, n = 3. Statistical analysis was performed within each condition through two-way ANOVA followed by Tukey’s multiple comparison test. P values are indicated. **c)** Heat map showing the change in expression of the top 10 most σ^D^ responsive genes as well as their expression upon treatment with EMB, INH, and RIF compared to uninduced WT using RNA-Seq. **d)** Volcano plot of differentially expressed genes between *M. abscessus* WT and the *ΔsigD* strain after 3h INH treatment, determined by RNA-Seq. The horizontal dashed line represents an adjusted p value of 0.01 and vertical lines represent a fold change of 1.5-fold. Genes with a positive fold change represent genes underexpressed in the *ΔsigD* mutant, while a negative fold change indicates genes overexpressed in the mutant. Genes with direct binding sites for σ^D^ determined by ChIP-Seq are indicated in magenta. Genes strongly dependent on σ^D^ are labeled.

While our studies thus far showed that σ^D^ is functional during growth in rich media, we wondered if perturbation of the cell envelope could further increase the expression the σ^D^ regulon. We therefore monitored the expression of *sigD* as well as MAB_1439c (strongly σ^D^ dependent and a proxy for σ^D^ activity) under conditions that perturb the cell envelope: SDS that induces cell wall stress, EMB that interferes with arabinogalactan synthesis and INH that interferes with mycolic acid biosynthesis [36,37]. Fig 4a shows that exposure to SDS modestly induced the regulon; EMB and INH caused ~2-fold increase in *sigD* transcript levels and a 5–10 fold increase in MAB_1439c levels; a similar induction was not observed in RIF treated bacteria. Moreover, the increase in transcript levels of MAB_1439c upon EMB and INH exposure was abrogated in a Mab*ΔsigD* mutant confirming that expression of MAB_1439c was σ^D^ dependent (Fig 4b). Consistent with this observation, induction of the σ^D^ regulon was identified in RNA-Seq of wild-type bacteria exposed to EMB and INH but not in RIF treated cells (Fig 4c, S1 Data) and [38]. Lastly, we compared the gene expression pattern in WT and Mab*ΔsigD* upon INH exposure which demonstrated that the induced expression of the σ^D^ regulon was abrogated in Mab*ΔsigD* (Fig 4d, S1 Data). Notably, the genes exhibiting σ^D^ - dependent expression under INH exposure largely overlapped with those identified under uninduced conditions, although the WT/Mab*ΔsigD* expression ratios were greater following INH treatment, consistent with induction of the σ^D^ regulon. Thus, INH exposure primarily increased the magnitude of σ^D^-dependent expression rather than recruiting a substantial number of additional genes into the regulon (S1 Table and S1 Data).

In *M. tuberculosis*, nutrient-starvation by transferring exponentially growing cells to phosphate buffered saline (PBS) leads to arrest in growth and respiration, yet bacteria remain viable and become phenotypically drug tolerant [32]. This state is associated with changes in lipid composition of the cell envelope, specifically accumulation of phthiocerol dimycocerosate (PDIM), and a remodeling of peptidoglycan involving L-, D- transpeptidase mediated enrichment in 3 → 3 crosslinks [32,39,40]**.** Curiously, three L-, D- transpeptidases were found to be direct targets of Mabσ^D^: MAB_1530, MAB_3165c and MAB_4537c. Since Mtb*sigD* is also known to be induced during nutrient starvation [32,41], we monitored the induction of MAB_1439c, a proxy for the σ^D^ regulon, under PBS starvation and in late stationary phase. As seen in Fig 4a, stationary phase growth and nutrient starvation in PBS resulted in an ~ 6-fold increase in transcription of Mab*sigD*, and an ~ 40–60 fold induction of MAB_1439c. Given the prominent induction of the σ^D^ regulon under starvation, we monitored the growth and survival of wild-type and Mab*ΔsigD* mutant during late stationary phase and in PBS; however, no significant difference in survival was observed between the strains under these conditions (S3 Fig).

### Induction of the σ^D^ regulon is controlled by RsdA degradation

**Fig 5:**
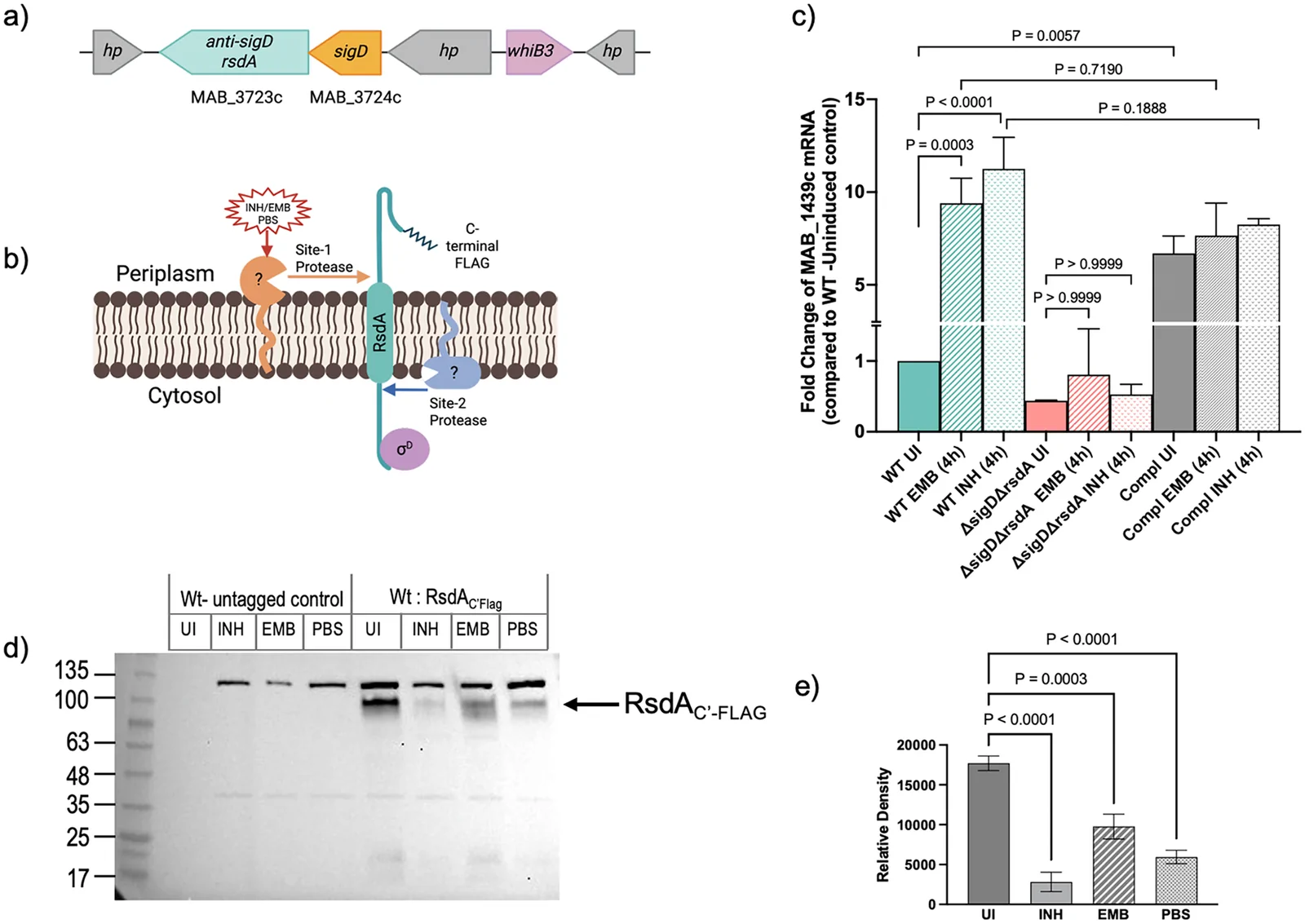
Induction of the σ^D^ regulon is controlled by RsdA degradation. **a)** Genomic organization of *sigD* and its adjacent anti-sigma factor *rsdA*. hp = hypothetical protein. **b)** Model of σ^D^ activation by intramembrane proteolysis of RsdA (Created in BioRender. Ghosh, P. 2026 https://BioRender.com/8vxlby1). RsdA is a membrane protein that anchors σ^D^ to the cell membrane. Activation of site-1 protease by cell wall stresses and nutrient starvation presumably cleaves RsdA at site1; subsequent cleavage by site-2 protease liberates functional σ^D^. RsdA was tagged at the C-terminal periplasmic domain with a 3X FLAG tag that enables monitoring its degradation under various conditions. **c)** Change in relative mRNA expression of MAB_1439c in WT, *ΔsigD/*Δ*rsdA* and *ΔsigD/*Δ*rsdA::*p_*sigD*_*sigD*_3’**-**FLAG_ strain when induced with EMB 70μg/mL or INH 64μg/mL for 4h measured using RT-qPCR and plotted as fold change over wild-type uninduced control. Data represents mean ± SD, n = 3. Statistical analysis was performed through one-way ANOVA followed by Tukey’s multiple comparisons test. **d)** WT untagged and Mab:: MAB_3723c_3’FLAG_ strains were grown either in PBS for 6h or exposed to EMB 70μg/mL or INH 64μg/mL for 6h followed by detection of RsdA_FLAG_ using anti-FLAG antibodies. Location of RsdA is indicated with an arrow; bands above and below RsdA are non-specific and present in the untagged (WT) strain as well. **e)** Quantification of RsdA from western blots by densitometric analysis using ImageJ. Data represents mean ± SD, n = 3. Statistical significance was determined by one-way ANOVA followed by Tukey’s multiple comparisons test; P values are indicated.

Alternate sigma factors are believed to be constitutively transcribed; their activity is however regulated post-translationally by anti-sigma factors that sequester them in an inactive state until activation is required. Anti-sigma factors regulating extracytoplasmic (ECF) sigma factors are often membrane proteins, encoded by genes adjacent to their cognate sigma factors genes on the chromosome. A putative anti-σ^D^/RsdA (MAB_3723c) lies immediately adjacent to Mab*sigD* in the *M. abscessus* genome (Fig 5a). Transmembrane topology prediction (DeepTMHMM 2.0) suggests a single-pass membrane protein with an N-terminal cytoplasmic sigma factor-binding domain and a C-terminal periplasmic domain, consistent with a role in sensing envelope status and relaying this signal through regulated intramembrane proteolysis, analogous to activation pathways described for *M. tuberculosis* RsdA, RskA, RslA, and RsmA [14,31] (Fig 5b). To determine if MAB_3723c functions as a regulator of σ^D^, we created a deletion of MAB_3723c in a Δ*sigD* mutant background (Δ*sigD*/Δ*rsdA*) and monitored the induction of MAB_1439c in the presence of EMB and INH. Fig 5c shows a lack of MAB_1439c expression in a Δ*sigD*/Δ*rsdA* strain as expected; in a Δ*sigD*/Δ*rsdA::*p_*sigD*_*sigD* strain background, induced expression levels of MAB_1439c was observed even in the absence of EMB and INH, confirming that RsdA controls the induced expression of the σ^D^ regulon.

To determine if the observed induction of the σ^D^ regulon is associated with degradation of MabRsdA, we introduced a 3X FLAG tag at the 3’-end of MAB_3723c at its native chromosomal location using recombineering. This generated a C-terminal, periplasmically oriented FLAG tagged MabRsdA protein. Although an N-terminal cytoplasmic tag would have been preferable to track the cleavage product of RsdA, this was not technically feasible because the open reading frames of MAB_3723c and MAB_3724c overlap. The resulting Mab::MAB_3723c_3’ FLAG_ strain was exposed to EMB, INH or PBS starvation for 6h, and the cleavage of RsdA was assessed by monitoring the abundance of RsdA _C-FLAG_ by western blotting using α-FLAG antibodies. As seen in Fig 5d-5e, RsdA levels decreased significantly following exposure to INH, EMB and nutrient starvation indicating that induction of the σ^D^ regulon is a consequence of RsdA degradation.

### The Mab*ΔsigD* mutant displays multi-drug sensitivity

**Fig 6:**
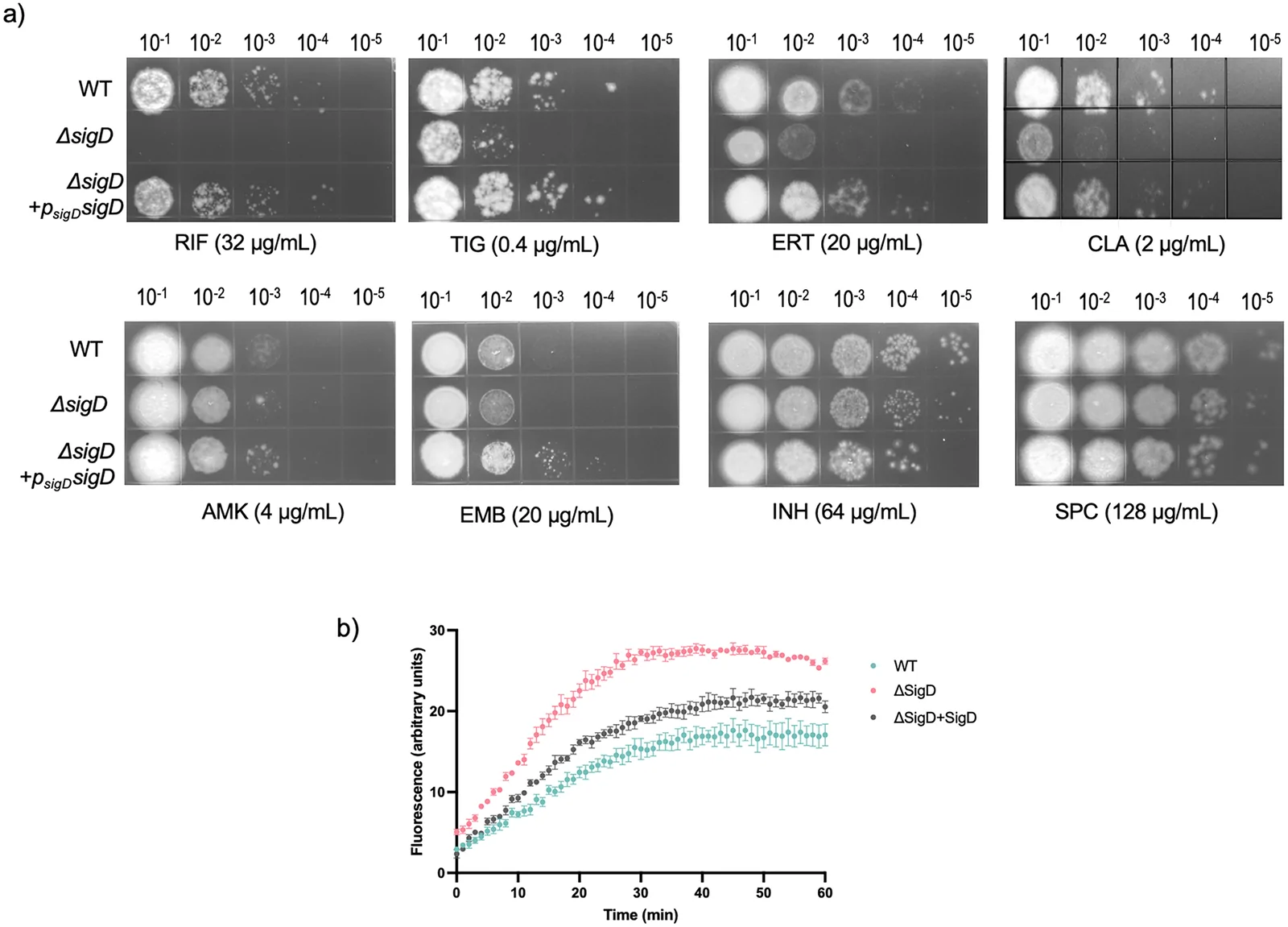
MabΔ*sigD* is sensitive to hydrophobic antibiotics and displays increased accumulation of ethidium bromide. a) Growth of ten-fold serial dilutions of WT, *MabΔsigD and MabΔsigD*::*p*_*sigD*_*sigD*_*C-FLAG*_ strains on Middlebrook 7H10 OADC containing indicated concentrations of RIF, TIG, ERT, CLA, AMK, EMB, INH, and SPC. Data is representative of >3 independent experiments. b) WT, *Mab*Δ*sigD and Mab*Δ*sigD*::*p*_*sigD*_*sigD*_*C-FLAG*_ strains were evaluated using the ethidium bromide (EtBr) uptake assay. Cells were incubated with PBST, 0.8% glucose, and 4 μg/mL EtBr and fluorescence was measured over the course of 1h. An increase in fluorescence is indicative of increased EtBr accumulation. Data represents mean ± SD, n = 3.

Nutrient-starved bacteria accumulate less antibiotics than replicating cells which cannot be reversed by efflux pump inhibitors indicating a reduced cell envelope permeability as a driver of starvation-associated tolerance [42]. Presumably, these changes are associated with cell envelope remodeling following transcriptional reprogramming by core stress regulators and sigma factors. Since the σ^D^ regulon constitutes several genes encoding cell wall associated proteins which are upregulated by starvation and envelope stress, we evaluated the sensitivity of the Mab*ΔsigD* strain to an array of antibiotics. As seen in Fig 6a, Mab*ΔsigD* was hypersensitive to RIF, TIG, clarithromycin (CLA) and erythromycin (ERT), but not to EMB, INH, AMK or spectinomycin (SPC) even though EMB and INH induce the σ^D^ regulon. We noticed that the antibiotic sensitivity pattern appeared to be a function of drug hydrophobicity (RIF > CLA/TIG/ERT > SPC > EMB/INH/AMK) rather than that of drug size (RIF > ERT/CLA > AMK/TIG > SPC > EMB > INH) and wondered if the observed hypersensitivity of Mab*ΔsigD* was suggestive of a compromised cell envelope that increased passive drug permeability. To test this, we monitored real-time ethidium bromide (EtBr) accumulation in wild-type, MabΔ*sigD*, and complemented strains over one hour. Indeed, Mab*ΔsigD* demonstrated a marked increase in EtBr accumulation compared to both the wild-type and complemented strains (Fig 6b).

**Fig 7:**
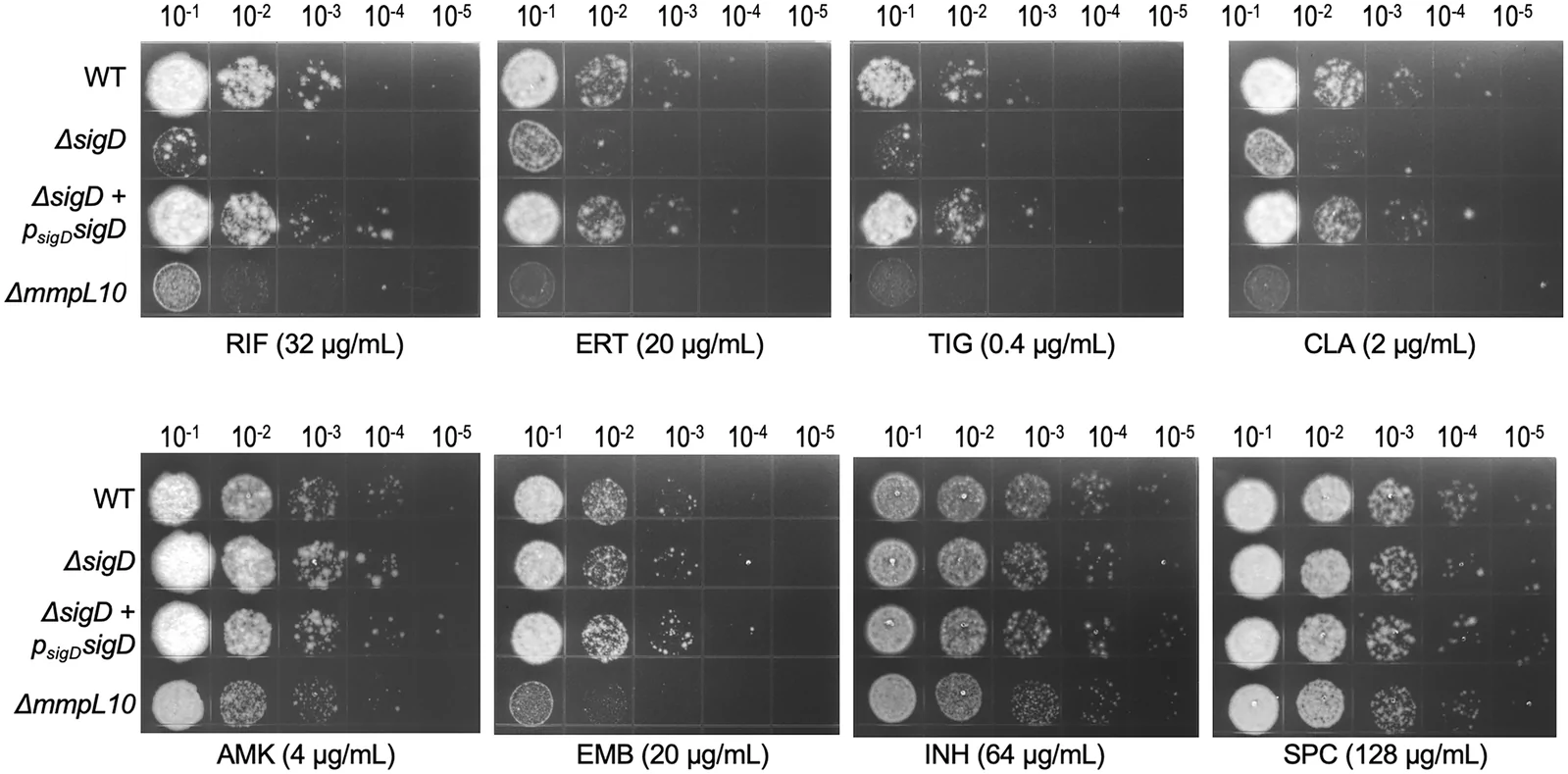
MabΔ*mmpL10* recapitulates the antibiotic sensitive phenotype of MabΔ*sigD.* Growth of ten-fold serial dilutions of *M. abscessus* WT, *Mab*Δ*sigD*, *Mab*Δ*sigD*::*p*_*sigD*_*sigD*_*C-FLAG*_ and Mab*ΔmmpL10* on Middlebrook 7H10 OADC containing indicated concentrations of RIF, TIG, ERT, CLA, AMK, EMB, INH, and SPC. Data is representative of >3 independent experiments.

Given the observed depletion of TPP in Mab*ΔsigD* we hypothesized that the antibiotic hypersensitivity of MabΔ*sigD* stems, at least in part, from reduced TPP levels in the cell envelope. We therefore constructed an isogenic deletion of *mmpL10*; the Δ*mmpL10* mutant was clearly hypersensitive to RIF, TIG and CLA suggesting that TPPs in the mycobacterial cell envelope may play a protective role against antibiotics (Fig 7).

## Discussion

σ^D^ is one of sixteen group IV extra-cytoplasmic (ECF) sigma factors in *M. abscessus* and is therefore appropriately poised to transduce signals from the cell surface or the extracellular environment. Here we define the σ^D^ regulon of *M. abscessus* and show that 447 genes are differentially expressed in a Mab*ΔsigD* strain relative to wild-type (>1.5-fold change, *p*_*adj*_ < 0.01) during exponential growth. Only a subset of this regulon (~50 genes) appears to be directly controlled by σ^D^ and we identify a strong consensus promoter motif (GTAACA/G-N_16_-CGAT). Among the direct targets are four transcription factors from the TetR and WhiB families which likely amplify the σ^D^ response through secondary regulatory cascades. Interestingly, several genes containing σ^D^ promoters in their upstream regions showed little or no reduction in expression in the MabΔ*sigD* mutant under either uninduced or INH-induced conditions. This suggests that these promoters can also be recognized by additional sigma factors, allowing transcription to be maintained when σ^D^ is absent. This apparent redundancy is consistent with the complex architecture of transcriptional regulation in mycobacteria, which encode an unusually large repertoire of sigma factors with partially overlapping promoter specificities and stress-responsive functions. In *M. abscessus*, the presence of approximately 18 alternative sigma factors provides multiple opportunities for compensatory promoter recognition, such that loss of a single sigma factor may have only a limited effect on transcription of genes. Indeed, overlap in promoter recognition among mycobacterial sigma factors has been demonstrated previously, supporting a model in which functional crosstalk between sigma factor regulons buffers gene expression against the loss of individual regulatory pathways [24,25,28,34].

Notably, more than half of the genes directly regulated by σ^D^ are cell envelope associated, including those that encode proteins involved in mycomembrane biosynthesis/transport, membrane associated proteins, secreted proteins of hitherto unknown function, and several peptidoglycan hydrolases and L,D- transpeptidases which mediate 3 → 3 crosslinks known to strengthen the cell wall during stress and starvation. In line with this expectation, nutrient starvation and agents that cause envelope stress (SDS) or disrupt envelope biosynthesis (INH, EMB) increased expression of the σ^D^ regulon via degradation of RsdA. Together these findings support a role for the σ^D^ regulon in cell envelope maintenance during exponential growth, and an induced expression during adaptation to perturbations at the cell surface.

Within the σ^D^ regulon, two loci were particularly striking - the TPP locus implicated in trehalose polyphleate (TPP) biosynthesis/transport, and the Ag85 locus involved in TDM and mAGP formation; of these TPPs have not been detected in the envelope of *M. tuberculosis*. Remarkably, the Mab*ΔsigD* strain showed a significant decrease in TPP levels without any detectable change in TDM content (Fig 3f). Furthermore, the amounts of diacyltrehalose precursors (DAT) in the mutant strain were indistinguishable from that of wild-type bacteria. This observation is consistent with the presence of a σ^D^ regulated promoter intragenic to MAB_0938c (*papA3*) and an ~ 4-fold decrease in expression of *mmpL10* which is known to be required for translocation of DATs to the cell surface where they are transacylated to phleic acids to form TPPs [43,44]. The reduced expression of *mmpL10* in Mab*ΔsigD* likely contributes to the observed differences in TPP.

Curiously, despite strong induction of the σ^D^ regulon during nutrient starvation and stationary phase, the Mab*ΔsigD* mutant exhibited no significant defect in survival under these conditions, suggesting that genes required for persistence in nutrient-limited environments are controlled by redundant regulatory pathways. Similarly, although EMB and INH induced the σ^D^ regulon, the susceptibility of Mab*ΔsigD* to these antibiotics was indistinguishable from that of wild-type bacteria. Instead, the most pronounced and reproducible phenotypes were observed with RIF, TIG, CLA and ERT, all of which are hydrophobic in nature. In contrast, antibiotics without detectable effects in our assay were generally more hydrophilic (AMK, EMB, INH, SPC). Furthermore, deletion of *sigD* also increased intracellular accumulation of ethidium bromide. Although net EtBr accumulation is a cumulative measure of both passive influx and active efflux, the deletion of the σ^D^ regulon, which heavily regulates envelope-associated genes, suggests that this increased accumulation is driven by changes envelope permeability, though a concurrent contribution of impaired efflux cannot be entirely ruled out.

Collectively, these findings suggest that loss of σ^D^ likely increases permeability to hydrophobic compounds, rather than altering susceptibility through a drug-specific target or pathway. This interpretation is supported by previous studies in *M. tuberculosis*, *M. smegmatis* and *M. marinum* which show that reduced mycolates are linked to increased cell envelope fluidity and greater permeability to hydrophobic antibiotics such as RIF [45,46] and that nutrient-starved bacteria accumulate less rifamycins, fluoroquinolones and linezolid than replicating cells and have a reduced cell envelope permeability [42]. However, because the mycolic acid content of the Mab*ΔsigD* mycomembrane was indistinguishable from that of wild-type bacteria, altered mycolate abundance is unlikely to account for the increased permeability observed here. Instead, the Mab*ΔsigD* envelope contained substantially lower levels of TPP and a deletion of *mmpL10*, which eliminates envelope-associated TPP, also increases susceptibility to the same hydrophobic antibiotics. Together these findings support a model in which TPP contributes to mycomembrane permeability and limits antibiotic penetration. Although an additional role for MmpL10 in drug efflux cannot be excluded, previous studies argue against this [19].

It is noteworthy that several genes regulated by Mabσ^D^ (MAB_0175, MAB_0177, MAB_0405c, MAB_1439c, MAB_1616, MAB_2871c and MAB_3355) have been previously associated with macrophage TLR2 activation [47], and an Mtb Δ*sigD* mutant has been reported to reduce macrophage TNF-α production [30]. Because TLR2 signaling promotes TNF-α production in macrophages, it is plausible that the *M. abscessus* Δ*sigD* mutant may similarly attenuate TNF-α responses. This possibility is particularly intriguing given the proposed role of TPPs in survival of *M. abscessus* in macrophages by inhibiting phagosome acidification [19]. Future work would involve testing if the σ^D^ regulon influences macrophage signaling and virulence phenotypes of MabΔ*sigD* in mouse models.

Finally, although the primary amino-acid sequence of Mabσ^D^ is ~ 74% identical to Mtbσ^D^, a comparison of their regulons reveals a limited overlap (15 genes) which includes *papA3*, *rpfC* and *ag85-C* (S2 Data). While all three genes are strongly dependent on σ^D^ in *M. tuberculosis*, only *papA3* shows a strong σ^D^ dependence in *M. abscessus*; *rpfC* and *ag85C* exhibit σ^D^ occupancy without detectable expression changes, again suggesting compensatory or redundant regulation. Additionally, the strong dependence of Mtbσ^D^ on *iniA* and *iniB* transcription is completely absent in *M. abscessus*; conversely a homologue of MAB_1439c, encoding a putative secreted protein in *M. abscessus* and reliant on Mabσ^D^, is absent in *M. tuberculosis*. Furthermore, the consensus sequence for Mabσ^D^ promoters identified here also differs from motifs described previously for Mtbσ^D^ by two groups: Raman et al reported σ^D^ dependent promoters in H37Rv strain to contain a clear -35 consensus of GTAACGct and no -10 consensus motif, whereas Calamita et al reported a consensus of AGAAAG-N_16_-20-CGTTAA in the CDC1551 strain [29,30], but is more likely a consequence of different methods employed in these studies (ChIP-Seq in *M. abscessus* v RNA-Seq in *M. tuberculosis*). Surprisingly, a comparison of the σ^D^ regulon of *M. abscessus* with the closely related actinobacterium, *Corynebacterium glutamicum* revealed more similarities than with *M. tuberculosis*. Overexpression of Cgu *sigD* induces expression of multiple genes involved in corynomycolic acid synthesis, corynomycolyl transferases and L,D-transpeptidases, and results in accumulation of trehalose dicorynomycolate (TDCM) in the envelope [48,49]. Additionally, the *C. glutamicum* σ^D^ consensus promoter sequence GTAACA/G-N_17-18_-GAT is nearly identical to that identified here for *M. abscessus* [50]. Despite the similarities in their regulons and promoter sequences, a change in mycolic acid content was not observed upon overexpression of Mab_*sigD* in Mab*ΔsigD* (Fig 4c)*.* This is not surprising given the essential nature of mycolic acids and cell envelope glycolipids in *Mycobacterium* spp. as compared to corynebacteria which necessitates the existence of redundant regulatory pathways for envelope lipid synthesis. Overall, the differences among mycobacterial σ^D^ orthologs in the gene sets they regulate illustrate how related transcriptional regulators can be evolutionarily repurposed across actinobacteria. While σ^D^ contributes to growth and virulence in *Mycobacterium tuberculosis*, our findings establish a role for Mabσ^D^ in cell-envelope maintenance; its potential contribution to host interactions and virulence remains to be determined experimentally.

## Materials and methods

### Media and bacterial strains

*M. abscessus* CIP104536^R^, an isogenic, glycopeptidolipid (GPL)-negative rough mutant derived from the wild-type ATCC19977 strain [18], and its derivatives were grown in Middlebrook 7H9 (DIFCO) supplemented with 0.05% Tween 80 and 10% OADC. Antibiotics were added at appropriate concentrations when indicated. Isogenic deletion of *MAB_3724c* (*sigD*) and MAB_0937c (*mmpL10*) were generated in the CIP104536^R^ strain using recombineering, followed by the removal of the apramycin cassette by Cre-mediated recombination at *loxP* sites as previously described [22]. A deletion of MAB_3723c was similarly created in a Δ*sigD* background. *MAB_3723c* was endogenously tagged with a C-terminal FLAG tag using recombineering to create Mab::MAB_3723c_3’FLAG_. Unmarked deletions were confirmed through PCR and sequencing of product (S1 Fig). Complementing strains were created by cloning a gene of interest into pMH94 under the control of either a constitutive promoter (*hp60*) or its native promoter followed by integration of the plasmid into the L5 *attB* site of the appropriate deletion background. Mab*sigD* was cloned with a 3’-3X FLAG tag, expressed from its native promoter and integrated into the L5 location of a MabΔ*sigD* strain- Mab*ΔsigD::*p_*sigD*_*sigD*_3’**-**FLAG_.

### Growth of bacteria under nutrient starvation

Bacterial strains under study were grown in Middlebrook 7H9 + OADC to A_600_ = 0.6. The cells were harvested, washed with four times the original volume of the culture of phosphate buffered saline (PBS) with 0.05% (vol/vol) Tyloxapol, as previously described [51]. The bacteria were resuspended in PBS with 0.05% (vol/vol) Tyloxapol and incubated at 37°C for the indicated duration of time (6h, 24h, 48h). Cells were removed and assayed as required either for viability, drug sensitivity or for western blotting.

### Antibiotic Sensitivity Assays

Wild-type, mutant, and complementing strains of *M. abscessus* were grown to A_600_ = 0.8. Cells were tested for drug susceptibility by spotting a 10-fold dilution series onto Middlebrook 7H10 agar supplemented with 10% OADC and the indicated concentration of antibiotic.

### Western Blotting

To follow the degradation of RsdA, the Mab:: MAB_3723c_3’FLAG_ strain was grown either in PBS for 6h or exposed to INH/EMB for 6h. The samples were normalized by A_600_ and weight, gently sonicated followed by separation of proteins on a 5–15% gradient SDS polyacrylamide gel (Biorad). Gels were either stained with Coommassie Blue or transferred to a PVDF membrane. RsdA_FLAG_ was detected using anti-FLAG antibodies (Invitrogen). 3 biological replicates were performed.

### RNA Preparation and RT-qPCR

Wild-type *M. abscessus*, MabΔ*sigD* and Mab*ΔsigD::*p_*sigD*_*sigD*_3’**-**FLAG_ were grown to exponential phase (A_600_ = 0.6) followed by treatment with 70μg/mL ethambutol (EMB), 64μg/mL isoniazid (INH), 32 μg/mL of rifampicin (RIF), or 0.05% SDS for indicated times. To study the effect of starvation, bacteria were grown to late stationary phase (A_600_- = 6.0) or washed with phosphate buffered saline (PBS) and incubated for an additional 6h. Total RNA was prepared using the Qiagen RNA preparation kit followed by TURBO DNase treatment. cDNA was generated using random hexamers and Maxima reverse transcriptase (ThermoFisher), and RT-qPCR was performed with the SYBR Green RT-qPCR Master Mix (ThermoFisher) using the following primer pairs: *sigD* 5’-CCGTTCCTGGCATTTGTGTA and 5’-ACGCGCAAGATCAGGATTTC; MAB_1439c- 5’-ATGGCGACGACGATGAC and 5’-AGTGGACCA

CGCCATTC. Applied Biosystems Quant Studio 5 Real-Time PCR System was used with cycling conditions: 50°C for 2 min, 95°C for 10 min, and 40 cycles of 95°C for 15 s, 60°C for 1 min. Relative transcript levels were determined using the comparative Ct (2^−ΔΔCt^) method. Ct values for each target gene were normalized to the endogenous control gene *sigA* (ΔCt), and relative fold changes were calculated by comparison with the indicated uninduced control (ΔΔCt). Data represents mean ± SD, n = 3.

### RNA-Seq analysis

Wild-type *M. abscessus*, Mab*ΔsigD* and Mab*ΔsigD::*p_*sigD*_*sigD*_3’**-**FLAG_ strains were grown to exponential phase (A_600_ = 0.6) and exposed to either 70μg/mL EMB, 32μg/mL RIF, or 64μg/mL INH when indicated. RNA extraction, DNase treatment, cDNA synthesis and library prep was performed by SeqCenter (Pittsburgh) and sequenced on an Illumina Novaseq platform. The sequence data was analyzed using the reference-based analysis and default parameters on Rockhopper v2.03. The raw counts generated were then analyzed using DESeq2 that reports differential gene expression and p_adj_ values. [52–54]. RNA-Seq experiments were performed using 2 biological replicates. Raw data for all RNA-Seq have been deposited in GEO (accession # GSE338978).

### Chromatin immunoprecipitation sequencing (ChIP-Seq) and data analysis

ChIP-Seq was performed as previously described with minor modifications [7,55]. The Mab*ΔsigD::*p_*sigD*_*sigD*_3’**-**FLAG_ strain was grown at 37°C in Middlebrook 7H9 supplemented with 10% OADC and 0.05% Tween 80 to an A_600_ of 0.6 followed by crosslinking with 1% formaldehyde for 30 min and quenching with 250mM glycine. Cells were lysed using the CryoMill (Retsch) followed by sonication for 30 mins using the Bioruptor sonicator (Diagenode). The DNA protein complex was immunoprecipitated with anti-FLAG monoclonal antibody M2 (Sigma) for 18h at 4°C and processed as previously described [7]. Each ChIP-Seq experiment was performed using two biological replicates. Genomic DNA libraries enriched for σ^D^ binding were prepared using the NEB NextUltra II Library Prep kit for Illumina followed by sequencing on the Illumina platform. Reads were aligned to the reference genome using Rockhopper v2.03. Regions of enrichment were identified using a custom Python script as described previously [7]. Relative enrichment is reported as fold above threshold (FAT) score. The enriched regions were analyzed using MEME Suite 5.5.9 using default parameters [56]. ChIP-Seq experiments were performed using 2 biological replicates. Raw data for all ChIP-Seq have been deposited in GEO (accession # GSE338978).

### Ethidium Bromide (EtBr) uptake assay

Ethidium bromide uptake assays were performed as previously described with minor modifications [57]. Wild-type, Mab*ΔsigD*, and Mab*ΔsigD::*p_*sigD*_*sigD*_3’**-**FLAG_ were grown to A_600_ of 0.8 washed in PBS containing 0.05% Tween 80 (PBST) and resuspended to an A_600_ of 0.8 in PBST. 100μL of each strain was mixed with 100μL of PBST supplemented with 0.8% glucose and 4μg/mL EtBr. A control sample lacking bacteria was also incorporated. Real-time fluorescence measurements were carried out over a time frame of 60 mins using a fluorescence spectrometer (Molecular Devices, SpectraMax M5), with excitation and emission wavelength set at 530 and 595 nm, respectively. Results were normalized by subtracting out fluorescence from the control wells. Experiments were performed using >3 biological replicates.

### Lipid extraction and Thin Layer Chromatography (TLC)

Wild-type *M. abscessus*, *ΔsigD*, *ΔmmpL10*, and complemented strains were grown overnight to A_600_ = 0.8, pelleted and wet cell weight was determined. Total free lipids, apolar lipids and mycolic acid methyl esters (MAMEs) were purified as previously described with minor modifications [19,58]. Total lipids were extracted from ~100 mgs of cell pellets treated successively with CHCl_3_/CH_3_OH (1:2)) and CHCl_3_/CH_3_OH (2:1), washed with dH_2_O and dried. Apolar lipids were extracted from ~100mg of cell pellets resuspended in MeOH:0.3% NaCl (100:10) through 2 additions of petroleum ether, and dried. For purification of crude MAMEs, ~ 100mg of cells were resuspended in 15% tetrabutylammonium hydroxide (TBAH) and heated at 105°C for 18h. The mixture was then diluted and mixed with dichloromethane and iodomethane. After mixing for 1h, the organic layer was washed 3 times with dilute HCl followed by 2 washes with dH_2_O and dried. Dried total lipids, apolar lipids and MAMEs were normalized by weight before resuspension in an appropriate solvent. Total lipids were resuspended in CHCl_3_; apolar lipids and MAMEs were resuspended in dichloromethane, and equal quantities were loaded on Silica gel 60 F_254_ TLC plates (Sigma Aldrich). Total lipids were separated using CHCl_3_/CH_3_OH (90:10 v/v) and visualized by spraying the plates with a 0.2% anthrone (Sigma, 90-44-8) solution (w/v) in concentrated H_2_SO_4_ and charring. Apolar lipids were resolved in a solvent system of CHCl_3_:MeOH:H_2_O (90:10:1 v/v) x2 and visualized by spraying with 10% H_2_SO_4_ in EtOH and charring at 120°C. MAMEs were resolved in a solvent system containing petroleum ether:diethyl ether (95:5 v/v) x6 and visualized by spraying 5% phosphomolybdic acid and charring at 110°C. Lipid purification and TLCs were performed using >5 biological replicates.

## Supporting information

S1 Fig Validation of mutant strains using PCR. Deletion of *sigD* and *mmpL10* were checked using the following primer pairs: 5’- GCGGCCGAAGAG. CTGGCG and 5’-TCCACCTGCGCGAGCTCG (sigD) and 5’-CGGAGCCGTCACGTATTACGG and 5’-TTCGGGAACCAGTCCTTGCC (mmpL10).
(PDF)

S2 Fig Functional analysis of untagged and 3XFLAG-tagged SigD. Growth of ten-fold serial dilutions of WT, *MabΔsigD, MabΔsigD*::*p*_*sigD*_*sigD* and *MabΔsigD*::*p*_*sigD*_*sigD*_*C-FLAG*_ strains on Middlebrook 7H10 OADC containing indicated concentrations of RIF. Data is representative of >3 independent experiments.
(PDF)

S3 Fig Survival of WT *M. abscessus* WT and *ΔsigD* during nutrient starvation. Survival of WT *M. abscessus* WT and *MabΔsigD* strains under nutrient starvation was assessed by a) incubation in PBS or b) growth to late stationary phase (A_600_ = 6.0). CFU/mL for each strain were measured at various timepoints by plating ten-fold serial dilutions on Middlebrook 7H10 OADC.
(PDF)

S1Table Comparison of fold changes in gene expression of the direct targets of σ^D^ between WT and Mab*ΔsigD* under uninduced and INH induced conditions. (PDF)

S1 Data Differential gene expression in WT and Mab*ΔsigD,* Mab*ΔsigD*::*p*_*sigD*_*sigD*_*C-FLAG*_ when grown in Middlebrook 7H9 + ADC+tween to logarithmic phase and when induced with INH and EMB determined using RNA-Sequencing (RNA-Seq). (XLSX)

S2 Data Comparison of the σ^D^ regulons of *M. abscessus* and *M. tuberculosis.* (XLSX)

S1 Raw Image a-b) Wt and Mab:: MAB_3723c3’FLAG strains were grown either in PBS for 6 h or exposed to EMB 70μg/mL or INH 64μg/mL for 6 h followed by detection of RsdAFLAG using anti-FLAG antibodies. Location of RsdA is indicated with an arrow; bands above and below RsdA are nonspecific and present in the untagged (WT) strain as well. Samples were normalized by OD, cell weight and coomassie blue staining (c-d).
(PDF)
