# Supplementary material for: The SigD regulon of Mycobacterium abscessus determines cell envelope composition and antibiotic susceptibility
Source: PLoS Genet. 2026 Sep 2;22(9):e1012286. doi: 10.1371/journal.pgen.1012286 (PMC13537577; doi:10.1371/journal.pgen.1012286)
Supplement: Supplementary file 1 [file pgen.1012286.s001.pdf]

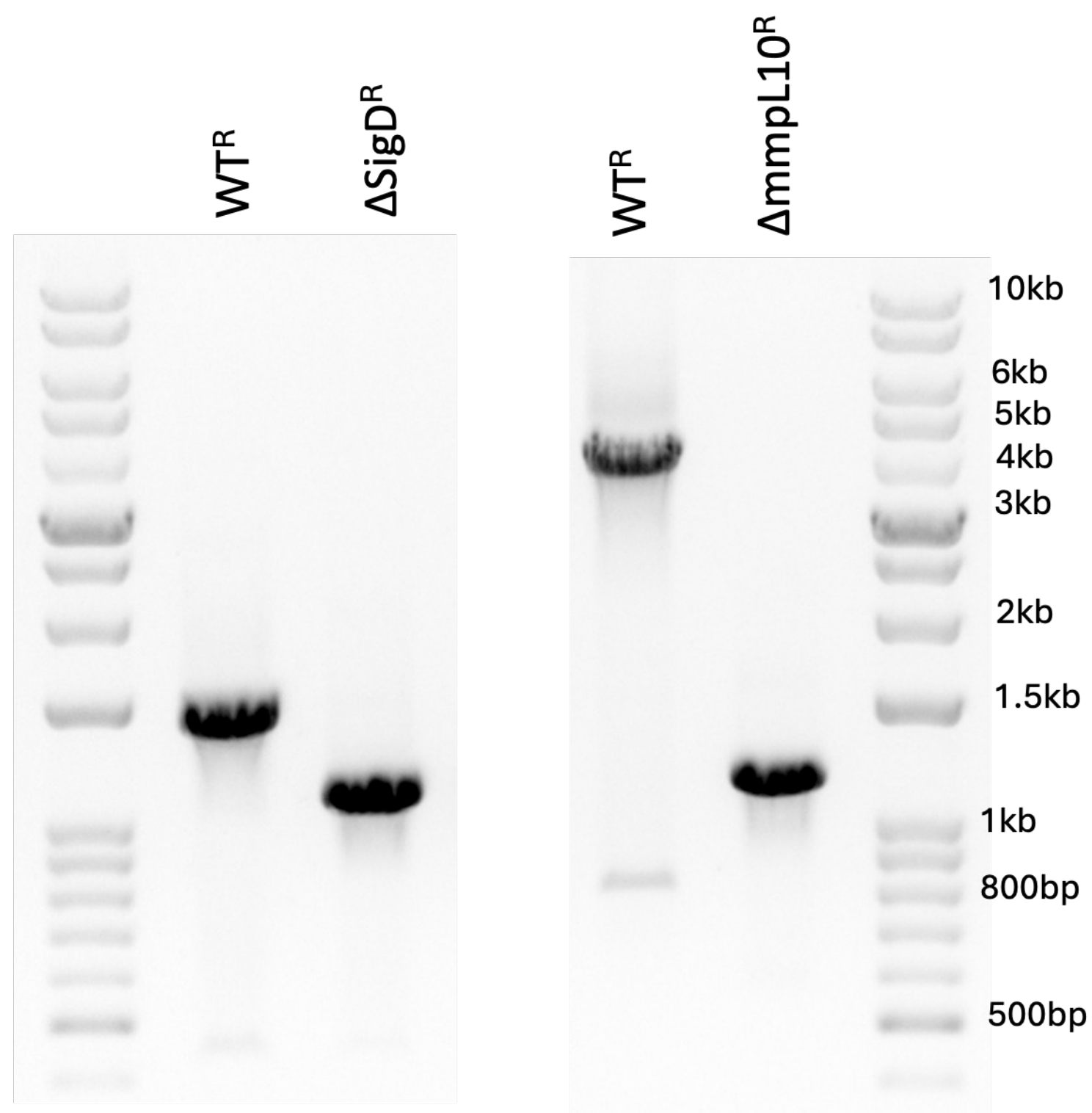

S1 Fig. Validation of mutant strains using PCR:

Deletion of *sigD* and *mmpL10* were checked using the following primer pairs: 5'- GCGGCCGAAGAGCTGGCG and 5'-TCCACCTGCGCGAGCTCG (sigD) and 5'-CGGAGCCGTCACGTATTACGG and 5'-TTCGGGAACCAGTCCTTGCC (mmpL10)
