# Supplementary material for: The SigD regulon of Mycobacterium abscessus determines cell envelope composition and antibiotic susceptibility
Source: PLoS Genet. 2026 Sep 2;22(9):e1012286. doi: 10.1371/journal.pgen.1012286 (PMC13537577; doi:10.1371/journal.pgen.1012286)
Supplement: Supplementary file 2 [file pgen.1012286.s002.pdf]

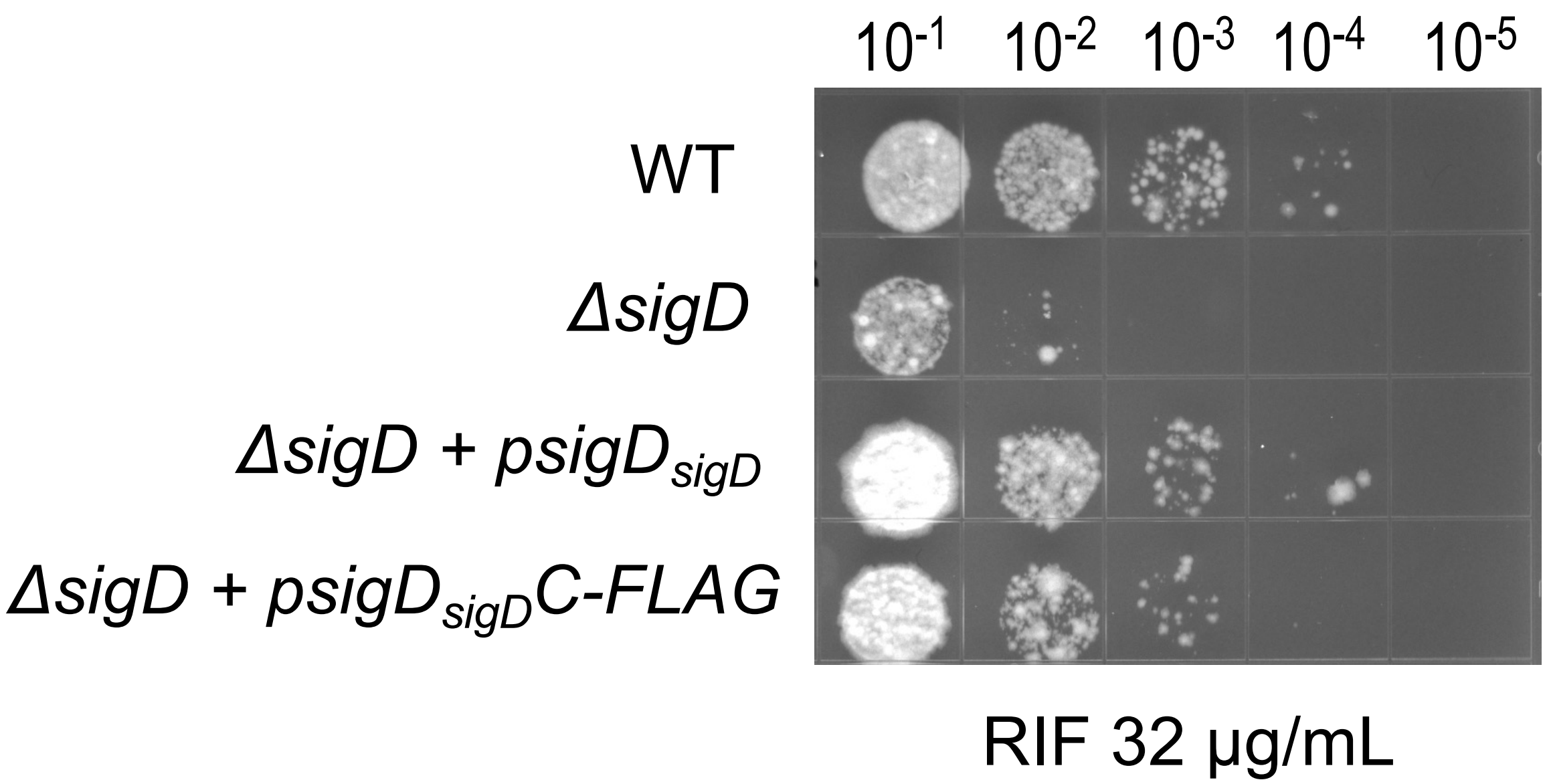

S2 Fig. Functional analysis of untagged and 3XFLAG-tagged SigD.
Growth of ten-fold serial dilutions of WT, *MabΔsigD, MabΔsigD*::p_{sigD}*sigD* and *MabΔsigD*::p_{sigD}*sigD*$_{C\text{-}FLAG}$ strains on Middlebrook 7H10 OADC containing indicated concentrations of RIF. Data is representative of >3 independent experiments.
