# Supplementary material for: The SigD regulon of Mycobacterium abscessus determines cell envelope composition and antibiotic susceptibility
Source: PLoS Genet. 2026 Sep 2;22(9):e1012286. doi: 10.1371/journal.pgen.1012286 (PMC13537577; doi:10.1371/journal.pgen.1012286)
Supplement: Supplementary file 3 [file pgen.1012286.s003.pdf]

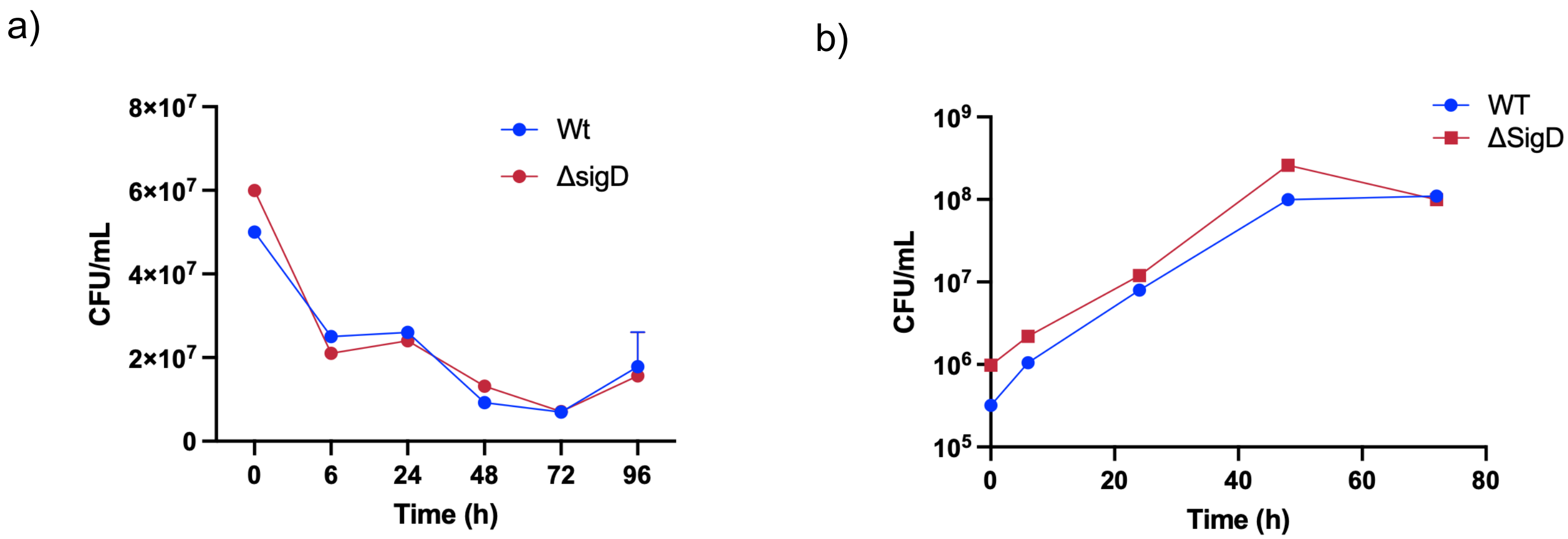

S3 Fig. Survival of WT *M. abscessus* WT and *ΔsigD* during nutrient starvation: Survival of WT *M. abscessus* WT and *Δ*Mab*sigD* strains under nutrient starvation was assessed by **a)** incubation in PBS or **b)** growth to late stationary phase (A_{600}=6.0) . CFU/mL for each strain were measured at various timepoints by plating ten-fold serial dilutions on Middlebrook 7H10 OADC.
