# Supplementary material for: The SigD regulon of Mycobacterium abscessus determines cell envelope composition and antibiotic susceptibility
Source: PLoS Genet. 2026 Sep 2;22(9):e1012286. doi: 10.1371/journal.pgen.1012286 (PMC13537577; doi:10.1371/journal.pgen.1012286)
Supplement: Supplementary file 4 [file pgen.1012286.s004.pdf]

S1 Table : Comparison of fold changes in gene expression of the direct targets of σ^D between WT and Mab*ΔsigD* under uninduced and INH induced conditions.

Gene	WT/dsigD	padj.WT_vs_dsigD	WT_INH/dsigD_INH	padj.WT_INH_vs_dsigD_INH
MAB_1439c	11.27	1.23E-78	17.10	4.75E-214
MAB_3724c	8.27	8.61E-43	42.07	8.57E-169
MAB_2488c	7.49	3.10E-62	7.92	1.09E-93
MAB_2961	5.49	1.27E-24	12.19	3.81E-68
MAB_2960	4.98	5.66E-54	8.56	2.05E-111
MAB_0938c	3.95	6.24E-26	9.83	1.87E-120
MAB_0937c	3.81	1.34E-36	5.96	1.59E-92
MAB_0733	3.79	7.72E-13	78.30	3.36E-75
MAB_4295c	3.57	1.84E-16	4.79	1.53E-12
MAB_4114	3.42	2.67E-31	6.30	1.49E-80
MAB_4296c	3.20	9.96E-33	6.10	1.44E-103
MAB_1152	2.62	7.25E-14	2.24	3.71E-13
MAB_1616	2.41	0.000123831	11.07	8.11E-53
MAB_1151	2.12	4.88E-07	2.19	6.98E-10
MAB_1150	2.08	0.001056229	0.98	0.96585749
MAB_3165c	2.03	3.58E-09	6.46	2.44E-82
MAB_1097	1.98	3.30E-05	1.00	0.997419096
MAB_4342c	1.97	0.000175681	1.06	0.895697055
MAB_2871c	1.97	3.16E-06	4.58	1.27E-46
MAB_2436	1.90	3.01E-06	4.59	1.45E-30
MAB_0409	1.84	1.50E-07	2.23	5.62E-11
MAB_1154	1.80	0.002232729	1.67	0.000714809
MAB_1153	1.79	0.000104413	1.82	8.59E-06
MAB_3206c	1.61	0.001149467	16.17	4.01E-117
MAB_1530	1.61	9.12E-05	3.24	2.51E-35
MAB_0405c	1.57	0.000290135	6.68	2.79E-67
MAB_4343c	1.47	0.427711423	2.62	0.038815117
MAB_0872	1.44	0.026708209	0.77	0.158065859
MAB_4620c	1.39	0.120845151	1.16	0.737397437
MAB_1149	1.37	0.010078764	1.34	0.0509583
MAB_0402	1.34	0.137918136	1.52	0.000182824
MAB_1118c	1.17	0.41628867	2.86	5.24E-18
MAB_3024	1.17	0.391669148	1.51	0.003906408
MAB_0039c	1.15	0.335128825	1.09	0.477698394
MAB_0062	1.15	0.379628914	0.99	0.940113488
MAB_3355	1.10	0.65163353	2.43	0.004729239
MAB_4154c	1.07	0.842308233	0.80	0.30614089
MAB_0175	1.06	0.762748117	2.73	1.10E-24
MAB_4080c	1.06	0.809933658	1.33	0.386786244
MAB_1512	0.98	0.916333592	0.99	0.981946294
MAB_1879c	0.95	0.813411083	0.79	0.021407404
MAB_3384c	0.93	0.702348409	0.82	0.203033922
MAB_2420c	0.84	0.407663769	1.08	0.65703435
MAB_2348	0.83	0.17265264	1.34	0.012386299
MAB_4537c	0.78	0.046872747	1.15	0.302399469
MAB_4230c	0.77	0.06317467	1.07	0.661784766
MAB_0706c	0.75	0.210498109	1.53	0.03830134
MAB_4528c	0.72	0.031614261	1.45	0.000186996
MAB_0017	0.68	0.092383519	1.48	0.027555654
MAB_0177	0.64	0.001008913	1.26	0.034363646
