# Supplementary material for: The SigD regulon of Mycobacterium abscessus determines cell envelope composition and antibiotic susceptibility
Source: PLoS Genet. 2026 Sep 2;22(9):e1012286. doi: 10.1371/journal.pgen.1012286 (PMC13537577; doi:10.1371/journal.pgen.1012286)
Supplement: Supplementary file 5 [file pgen.1012286.s007.pdf]

Legends for Raw images :

a-b) Wt and Mab:: MAB_3723c3'FLAG strains were grown either in PBS for 6 h or exposed to EMB 70µg/mL or INH 64µg/mL for 6 h followed by detection of RsdAFLAG using anti-FLAG antibodies. Location of RsdA is indicated with an arrow; bands above and below RsdA are non-specific and present in the untagged (WT) strain as well. Samples were normalized by OD, cell weight and coomassie blue staining (c-d).

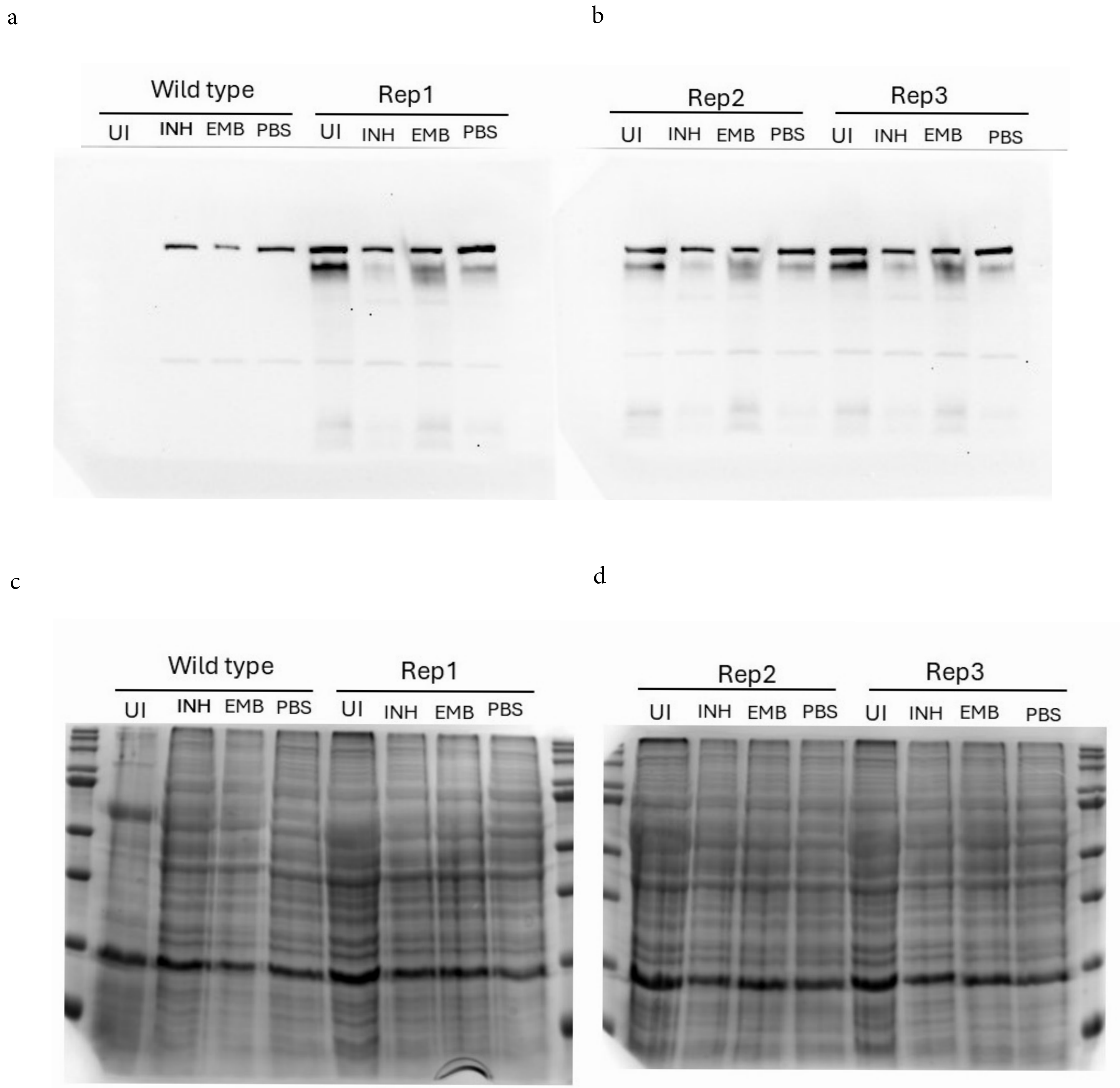

a-b) Wt and Mab:: MAB_3723c3'FLAG strains were grown either in PBS for 6 h or exposed to EMB 70µg/mL or INH 64µg/mL for 6 h followed by detection of RsdAFLAG using anti-FLAG antibodies. Location of RsdA is indicated with an arrow; bands above and below RsdA are non-specific and present in the untagged (WT) strain as well. Samples were normalized by OD, cell weight and coomassie blue staining (c-d).
